# Bereavement interventions for families in the ICU: a scoping review informed by a core outcome set

**DOI:** 10.1186/s13613-025-01557-6

**Published:** 2025-09-25

**Authors:** Sarah Foran, Mah Rukh, Alison Knapp, Jennifer M. O’Brien, Carol Brons, Janelle Glessman, Joann Kawchuk, Donna Goodridge, Sabira Valiani

**Affiliations:** 1 Department of Anesthesiology, College of Medicine, University of Saskatchewan, Saskatoon, SK Canada; 2 College of Medicine, University of Saskatchewan, Saskatoon, SK Canada; 3 Patient Family Partner, Saskatchewan Health Authority, Saskatoon, SK Canada; 4 Department of Medicine, College of Medicine, University of Saskatchewan, Saskatoon, SK Canada; 5 Department of Medicine, Department of Anesthesiology, College of Medicine, University of Saskatchewan, Saskatoon, SK Canada

**Keywords:** Bereavement, Grief, Death, Counselling, Social support, Critical care, Intensive care unit

## Abstract

**Background:**

Families confronting the death of a loved one in the intensive care unit (ICU) are at greater risk of experiencing complicated grief than families bereaved in other circumstances. The primary objective of this scoping review was to identify interventions that have been explored in the literature for supporting ICU bereaved families. Secondary objectives were to map the findings to a core outcome set (COS) developed to measure bereavement interventions for adults, and to identify existing knowledge gaps, with particular attention to considerations of equity, diversity, and inclusion (EDI).

**Methods:**

We searched five electronic databases: Web of Science, CINAHL, EMBASE, APA PsycInfo, and MEDLINE. Primary research articles that described bereavement program(s) and/or support(s) for bereaved loved ones in the setting of an adult ICU were included. We extracted data on study aims, methods, setting, patient and bereaved loved one demographics, design, analysis, and results. Four reviewers independently screened references and performed data extraction.

**Findings:**

We identified 11 bereavement interventions, including memorial services, condolence letters/sympathy cards, mementos, ICU diaries completed by staff or family, storytelling interventions, personalized/individual final wishes, information booklets/referrals to resources, meetings with ICU professionals, follow up telephone calls, training for staff, and other specific interventions. Most studies (35 out of 39) reported outcomes that aligned with a previously developed COS. Only three studies addressed cultural diversity in the development or implementation of bereavement interventions.

**Conclusions:**

This scoping review summarizes the range of bereavement interventions described in the literature and highlights key areas for future development, including cultural inclusivity in the development and implementation of interventions, and the use of standardized outcomes for evaluation.

**Registration:**

The protocol is registered on the Open Science Framework (https://osf.io/ue7t9).

**Supplementary Information:**

The online version contains supplementary material available at 10.1186/s13613-025-01557-6.

## Introduction

Bereavement after the death of a loved one is a universal experience that may be accompanied by physical, psychological, and social challenges [1]. While most individuals adapt over time, a subset experiences prolonged or complicated grief, characterized by intense, persistent symptoms that impair functioning [1–3]. Among families bereaved in the intensive care unit (ICU), the prevalence of complicated grief may be as high as 46–52% [4, 5].

Supporting bereaved ICU families is both a recognized responsibility and a gap in current ICU practice and literature [6–9]. Existing guidelines include only a weak recommendation to offer a written bereavement brochure as a means to reduce family anxiety, depression, and post-traumatic stress and improving family satisfaction with communication [10]. Globally, bereavement support varies widely in availability, timing, delivery, and provider [11, 12]. Most ICU bereavement literature has focused on pathological outcomes such as complicated grief and related comorbidities [13–16]. Two systematic reviews have found weak evidence to support any intervention, highlighting the need for culturally competent, locally informed approaches [12, 17].

Importantly, bereavement support extends beyond preventing pathology. A core outcome set (COS) for adult palliative care identifies two key outcomes: ‘Ability to cope with grief’ and ‘Quality of life and mental wellbeing’ [18]. However, randomized trials of bereavement interventions have incompletely addressed these core outcomes and often lack attention to equity, diversity, and inclusion (EDI) considerations [17]. Qualitative outcomes of ICU bereavement programs remain unmapped to the COS.

## Objectives

The objectives of this scoping review were to (1) identify the interventions for supporting ICU bereaved families that have been reported in the literature; (2) map the findings to a COS developed by Harrop and colleagues [18]; and (3) assess existing gaps in the literature, with particular attention to EDI considerations. To guide these objectives, the following research question was formulated: *What interventions for supporting ICU bereaved families have been reported in the literature*,* and what are the associated outcomes?*

## Methods

### Protocol and registration

This scoping review was conducted in accordance with the guidelines provided by the Preferred Reporting Items for Systematic Reviews and Meta-Analyses extension for Scoping Reviews (PRISMA-ScR) [19], together with recommendations provided by Arksey and O’Malley [20] and the Joanna Briggs Institute methodology for scoping reviews as expanded by Pollock and colleagues [21]. The protocol is registered on the Open Science Framework (https://osf.io/ue7t9) [22].

### Patient family partner involvement

This project was initiated by two Patient Family Partners (PFPs) with lived experience of ICU bereavement. Recognizing bereavement care as a major unmet need in their own experiences, they highlighted this gap to our research team and helped shape the study’s objectives, design (e.g. refining inclusion criteria, reviewing the core outcome set for relevance to bereavement in the ICU), and interpretation, ensuring alignment with the real-world needs and values of bereaved families. They participated in monthly virtual meetings throughout the project duration.

### Information sources

Studies were identified through searches of CINAHL, APA PsycInfo, Web of Science, MEDLINE, and EMBASE from inception to July 3, 2025, using a search strategy developed and peer-reviewed by a health sciences librarian at the University of Saskatchewan (Supplemental Material). There were no date restrictions, although studies without either an English or French full text were excluded. Primary research articles were included, whereas studies with only abstracts or conference abstracts available, as well as letters to the editor, reviews, syntheses, systematic reviews and meta-analyses, were excluded.

### Eligibility criteria

We included studies set within the ICU that reported on bereavement programs and/or supports directed to bereaved family members or loved ones. We excluded studies involving pediatric and neonatal ICUs, deaths occurring after discharge from the ICU, long-term ventilator-dependent patients, programs directed at healthcare providers, environmental scans (i.e., studies aimed at broadly mapping existing services, policies, or resources without evaluating or describing specific interventions), and studies focused on impacts to healthcare systems, resources, or providers. Long-term ventilator-dependent patients were excluded because their care typically extends beyond the acute ICU setting and reflects a distinct clinical course and family experience, as described in the literature on chronic critical illness [23, 24]. We also excluded studies that did not report or measure outcomes relevant to bereaved family members/loved ones, as well as those that were incomplete or only presented study protocols. Outcomes were considered relevant if they aligned with at least one domain from the COS for bereavement research, or if they reflected emotional, psychological, or social impacts of the intervention on the bereaved [18].

### Study selection

We imported studies obtained through the search strategy into the Covidence software, and duplicates were eliminated by the software and, if necessary, manually by reviewers. Four reviewers (S.F., M.R., J.O., D.G.) independently screened titles using the predefined inclusion and exclusion criteria. Articles passing the title and abstract screening stage were included for full text review, which was conducted independently by two of three reviewers (S.F., J.O., D.G.). We resolved conflicts from the title and abstract screening and full text review through discussion and mutual agreement among reviewers.

### Data extraction

Three reviewers (S.F., D.G., J.O.) independently extracted data using a data extraction tool created by the research team. Each extracted reference was assessed by a second reviewer. Conflicts in the data extraction process were resolved through discussion among the three reviewers. Data extracted from the studies included the study population, details of the bereavement intervention, outcome(s) measured, assessment tool(s), follow-up time point(s), and study findings.

### Data synthesis

We conducted a narrative description of the results for each study and categorized the interventions using the framework developed by Schut et al., extrapolated from the public health literature [25, 26]. This framework identifies three levels of intervention: universal, selective, and indicated. In bereavement care, these three levels correspond to Tier 1 (broad support and information), Tier 2 (targeted non-specialist support for those who are at risk of developing complex needs), or Tier 3 (specialist interventions for those with higher needs), respectively.

To enhance comparability across studies, we categorized reported outcomes as positive, neutral, or negative, based on the overall direction and tone of the study’s findings regarding bereavement interventions. A positive outcome was defined as either (1) a statistically significant improvement in at least one bereavement-related domain (e.g., reduced grief, higher family satisfaction, or improved psychological well-being), or (2) a clearly described improvement in the absence of statistical testing, such as consistent qualitative reports of benefit or author-identified improvements supported by participant feedback. In these cases, we relied on explicit descriptions within the results or discussion sections indicating a meaningful perceived benefit by participants or the study team. A neutral outcome indicated that the study reported no significant effect, mixed results without a clear direction, or insufficient detail to determine impact. A negative assessment was assigned when the intervention was associated with worsened outcomes or when participants expressed distress, dissatisfaction, or harm linked to the intervention. This categorization approach is consistent with prior scoping and narrative reviews where formal meta-analysis was not feasible due to heterogeneity in study designs, populations, and outcome measures [26].

In cases where studies evaluated multiple interventions or outcomes, each component was assessed independently, and the overall classification was based on the primary or most prominently reported findings. These distinctions are reported in the Supplementary Material to promote consistency and transparency. The outcomes were subsequently mapped to the COS developed by Harrop et al. [18]. The COS includes two core outcomes and nine associated thematic dimensions: (1) Ability to cope with grief (negative and overwhelming grief; communication and connectedness; understanding, accepting and finding meaning in grief; finding balance between grief and life going forwards; accessing appropriate support), and (2) Quality of life and wellbeing (participation in work and/or regular activities; relationships and social functioning; positive mental wellbeing and negative mental and emotional state).

### Findings

**Fig. 1:**
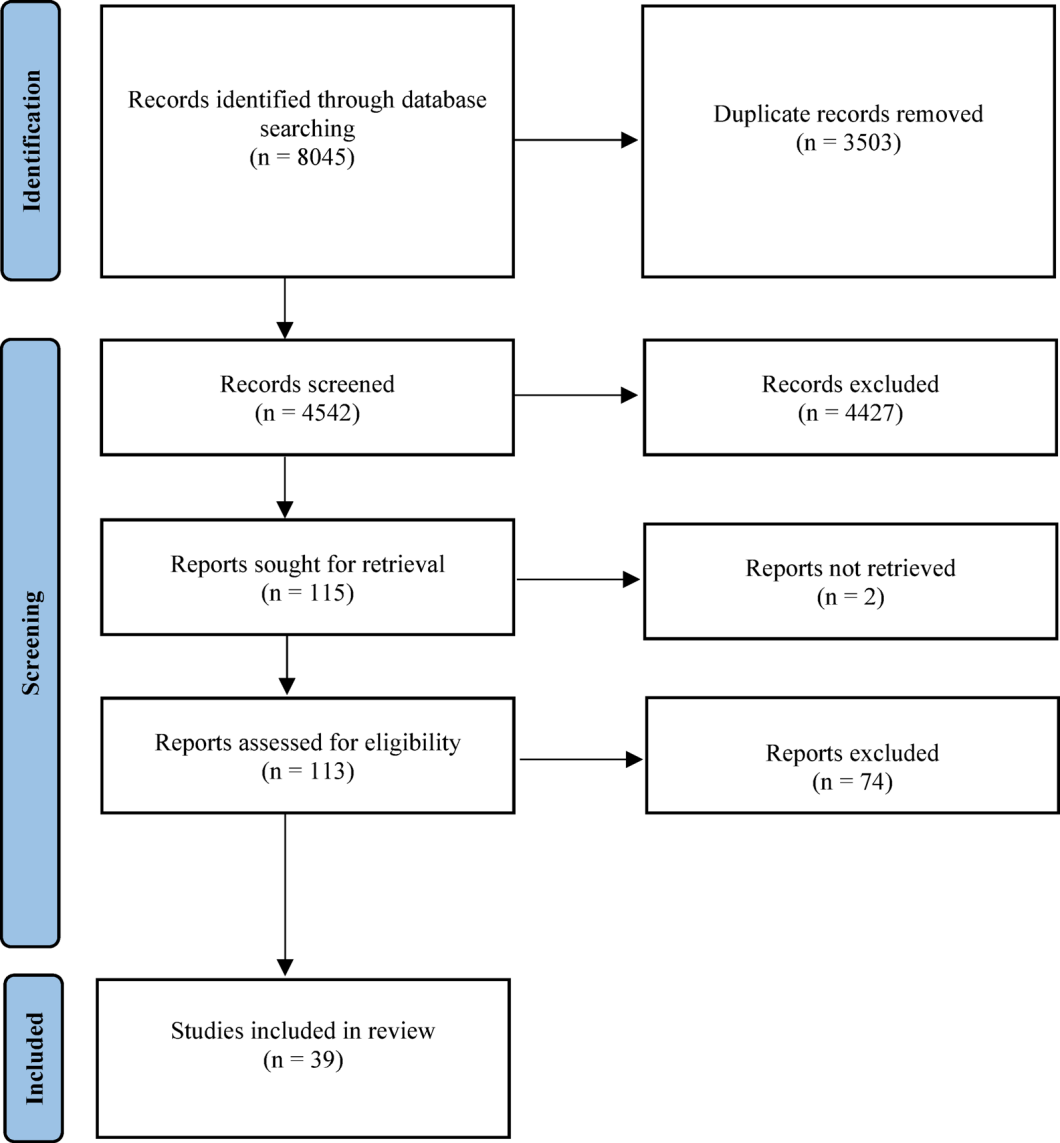
PRISMA flow diagram [19]

Searches identified 1,855 unique sources, of which 115 were deemed relevant based on title and abstract screening. We excluded 76 additional studies for reasons such as wrong or absent study design, intervention, outcomes, population, setting, or lack of an English full text. Following full-text review, 39 articles were included. A diagram of the study selection process is. shown in Fig. 1.

Of the studies, 18 were conducted in Canada/USA [27–44], 17 in Europe [14, 16, 45–59], and four in Australia [60–63]. Most (*n* = 23) studies employed qualitative methodology [28, 29, 32, 36, 37, 40–44, 46–49, 51–55, 57–59, 62], while others included three RCTs [14, 16, 63], one cohort study [27], and six pilot studies (including a single-blind trial, a prospective cross-sectional study, two observational studies, a single-arm pilot trial, and a qualitative descriptive study) [30, 33, 34, 38, 50, 56].

Details of the individual study characteristics and a summary of the bereavement interventions, including their overall assessments, are provided in the Supplementary Material. Several interventions were multi-component, and some studies fit into multiple categories. One study that evaluated a condolence letter noted increased symptoms of depression and post-traumatic stress disorder (PTSD) in the intervention group [16]. Another, assessed as neutral, noted that some relatives experienced ambivalence and felt a social obligation to respond to the letter [47]. A study that called bereaved relatives to offer emotional support and invite them to a follow-up meeting found that most declined the invitation; however, 42% of those who declined expressed appreciation for the call, reflecting mixed effects [56]. Diaries completed by staff or families were assessed as neutral in the majority of studies (3/5, 60%). While diaries were noted to support coherence and understanding, they also elicited strong emotional reactions, particularly when photographs were included. In some cases, families felt distressed upon viewing these images. Diaries requiring family input were also not always completed [48, 50, 60].

In 35 of the 39 studies, findings mapped to the COS established by Harrop et al., particularly within domains associated with the outcome ‘Ability to cope with grief’. These included negative and overwhelming grief [14, 16, 30, 33, 44, 45, 48, 50, 58], communication and connectedness [28–30, 37–44, 46–48, 54, 57, 61], understanding, accepting and finding meaning in grief [29, 30, 36, 37, 39, 41, 46–48, 50, 52, 55, 57, 59, 62], finding balance between grief and life going forwards [29, 37, 48, 54, 55], and accessing appropriate support [30, 49, 51–53, 59, 61]. Less frequently, participant outcomes pertaining to ‘Quality of life and mental wellbeing’ were reported in the dimensions of relationships and social functioning, positive mental wellbeing [29], and negative mental and emotional state (Table 3 of the Supplementary Material) [14, 16, 30, 33, 34, 39, 56, 60, 63].

Additionally, the bereavement intervention(s) in 24 of the 39 studies were categorized as Tier 1 (broad support and information), 14 as Tier 2 (targeted non-specialist support for those at risk of developing complex needs), and only one as Tier 3 (structured therapeutic follow-up by mental health professionals) [56].

We analyzed the inclusion of EDI considerations. Several studies collected demographic characteristics of equity-deserving groups, including sex [14, 16, 29–31, 33, 34, 44, 45, 47, 55–57, 59, 61–63], age [14, 16, 29–31, 33, 34, 44, 45, 47, 55–57, 59, 61–63], education level [14, 33, 34, 56], work/student status [14, 49, 60], language [31], religion [31, 33], and race/ethnicity [29–34, 39, 44, 60, 63]. Three studies addressed cultural diversity in the development and implementation of a bereavement intervention [33, 42, 43]. Two studies described culturally sensitive adaptations: one developed end-of-life training for healthcare professionals including cultural considerations and family discussions to help guide the bereavement process [43]; the other implemented a ‘grieving cart’ with multilingual religious resources and refreshments, checking permission with families to ensure cultural appropriateness [42]. A third study noted the ability to make cultural adaptations but did not specify what changes were made [33].

## Discussion

In this scoping review, we described interventions offered to bereaved families and found that most ICU-based bereavement interventions were perceived as beneficial by study investigators, with fewer studies reporting neutral or negative responses. Memorial services, mementos, storytelling, personalized final wishes, meetings with ICU healthcare providers, and staff training emerged as interventions generally reported as helpful to bereaved loved ones [14, 27, 29–31, 35, 36, 39, 41–44, 49, 52–54, 59, 62].

Interventions with mixed reviews included condolence/sympathy cards, diaries, and follow-up phone calls [16, 45, 47, 48, 50, 60, 63]. In studies reporting negative outcomes, investigators suggested these may have been related to the unexpected receipt of follow-up or the evocation of painful memories. Diaries, for example, were occasionally considered unhelpful—particularly when they included photos of the deceased or required active input from family members, which was not always feasible [48, 50, 60]. For follow-up telephone calls, two studies reported a mix of positive and negative responses [45, 47], and one study reported an overall neutral outcome [63]. In contrast, six other studies described overall positive outcomes [28, 34, 37, 38, 51, 61].

Bereavement care interventions have also been studied in the palliative care setting. A systematic review in that context identified a wide range of interventions—from pharmacotherapy to support groups and counseling—with no consistent evidence of benefit [64]. Participation in bereavement groups yielded mixed results, likely due to variations in delivery format, number of sessions, and group size. Notably, some bereaved family members viewed the palliative care team involved before death as an important source of support, suggesting a potential role for anticipatory or pre-emptive bereavement care [65].

Importantly, the European Society of Intensive Care Medicine (ESICM) published evidence-based and expert-informed guidelines in 2024 on end-of-life and palliative care in the ICU [66]. These guidelines recommend structured family conferences both before and after a patient’s death and advise that bereavement resources—such as brochures and informational leaflets—be integrated into these meetings. They also advise against the routine use of condolence letters from healthcare teams, although this recommendation is based on low-quality evidence. Overall, these recommendations are consistent with our review findings, which showed generally positive perceptions of meetings between healthcare teams and bereaved families, and mixed outcomes for condolence or sympathy cards.

Most studies in our review measured outcomes that mapped to the ‘Ability to cope with grief’ domain in Harrop’s core outcome set (COS). Fewer studies reported outcomes aligned with the ‘Quality of life and mental wellbeing’ domain, and those that did primarily reflected the subdimension of ‘negative mental and emotional state’ [14, 16, 30, 33, 34, 39, 60] This may reflect a tendency in bereavement research to measure symptoms such as depression and anxiety using tools like the Patient Health Questionnaire-9 (PHQ-9), Subjective Units of Distress Scale (SUDS), and the Hospital Anxiety and Depression Scale (HADS), rather than tools capturing resilience or positive wellbeing. These instruments are also commonly used in studies of Post-Intensive Care Syndrome – Family (PICS-F) [67].

Our Patient Family Partners (PFPs) played a central role in shaping the review methodology, reviewing the COS by Harrop et al. for relevance to the ICU context, and participating in mapping the identified outcomes. Their insights, grounded in lived experience and broader community engagement, helped define what a meaningful and compassionate bereavement intervention might look like. While the findings of this scoping review are intended to inform ICU bereavement practices broadly, they will also directly support the development of a regionally tailored bereavement support program in Saskatchewan. This includes integration into Cadence, a digital platform that offers grief and estate planning support [68]. Though specific to our setting, this implementation illustrates how evidence can be applied in a specific health system context. Strengths of this review include the use of a comprehensive, well-established scoping review methodology [19, 20], and the integration of PFPs throughout the research process. Our work is one of the few reviews to map bereavement intervention outcomes to the COS developed by Harrop et al., and while our implementation efforts will begin locally, the review’s findings and methodological approach are broadly applicable to practice and research. Unlike many prior studies that adopt a medicalized or pathological lens in evaluating grief [26] our review emphasizes a patient-centered, holistic approach.

Limitations include challenges in mapping outcomes to the COS, particularly due to overlapping constructs between domains (e.g., coping vs. quality of life), the subjectivity of categorizing qualitative data, and variability in how grief-related outcomes were defined and reported. Some classifications required multi-reviewer discussion to reach consensus. Importantly, evaluations were often based on investigators’ impressions rather than quantitatively measured or systematically analyzed using established qualitative methodologies, which limits the validity and reproducibility of findings.

Additionally, by excluding studies not published in English or French, our review may have missed culturally specific interventions developed in non-Western contexts. Most studies also lacked explicit integration of EDI considerations. Addressing these gaps will require future work that engages families from diverse backgrounds to enhance cultural relevance and accessibility. The ESICM guidelines emphasize the importance of tailoring end-of-life care to the cultural needs of patients and their families [66]. Other limitations include the heterogeneity in study designs, variations in measured outcomes, and a relative lack of quantitative data.

Future research should evaluate bereavement interventions using standardized outcomes, such as those outlined in the COS, and explore transitional models of care that bridge institutional and community-based support [69]. Furthermore, future research might investigate how the intensity of the intervention (Tier 1–3) and EDI factors influence outcomes. Ongoing collaboration with PFPs will be essential to ensuring bereavement programs are compassionate, comprehensive, and tailored to the diverse needs of families experiencing loss in the ICU.

## Conclusions

This scoping review identified a range of ICU bereavement interventions—including memorial services, keepsakes, storytelling, follow-up communication, and staff training—that were generally perceived as beneficial by study investigators. Outcomes most often mapped to ‘Ability to cope with grief’, with fewer addressing ‘Quality of life and mental wellbeing.’ Key gaps included limited attention to cultural diversity, inconsistent outcome measures, and absence of Tier 3 interventions. Together, these findings highlight priorities for future research and can inform the development of more comprehensive, culturally responsive ICU bereavement programs.

## Supplementary Information

Supplementary material 1.

## Data Availability

Not applicable.

## Abbreviations

ICU: Intensive care unit
COS: Core outcome set
EDI: Equity, diversity, inclusion
CCU: Coronary care unit
PRISMA-ScR: Preferred Reporting Items for Systematic Reviews and Meta-Analyses extension for Scoping Reviews
OSF: Open Science Framework
RCT: Randomized controlled trial
PFP: Patient Family Partner
ESICM: European Society of Intensive Care Medicine
PHQ-9: Patient Health Questionnaire-9
SUDS: Subjective Units of Distress Scale
HADS: Hospital Anxiety and Depression Scale
PICS-F: Post-Intensive Care Syndrome – Family
SCPOR: Saskatchewan Center for Patient Oriented Research
STEP: SCPOR Trainee Engagement with Patients
