# Supplementary material for: Bereavement interventions for families in the ICU: a scoping review informed by a core outcome set
Source: Ann Intensive Care. 2025 Sep 25;15:146. doi: 10.1186/s13613-025-01557-6 (PMC12463810; doi:10.1186/s13613-025-01557-6)
Supplement: Supplementary file 1 — Supplementary material 1. [file 13613_2025_1557_MOESM1_ESM.docx]

Interventions to Support Families through Bereavement in the ICU: A Scoping Review

Table of Contents

[Table 1: Characteristics of Included Studies 2](#_Toc198060730)

[Table 2: Bereavement Interventions (Brief) 12](#_Toc198060731)

[Table 3: Summary of Findings Mapped to Core Outcome Set 14](#_Toc198060732)

[eSupplement: Bereavement Interventions (Full) 16](#_Toc198060733)

[eSupplement: Database Search Strategies 49](#_Toc198060734)

[References 54](#_Toc198060735)

# Table 1: Characteristics of Included Studies

| **Source** | **Country** | **Study Aim** | **Study Design** | **Setting** | **Participant Demographics** | **Conducted in-hospital intervention component** | **Conducted out-of-hospital intervention component** |
| --- | --- | --- | --- | --- | --- | --- | --- |
| Akgün KM, et al. 2019 | United States | Compare the number of family meetings and scores on the Bereaved Family Survey-Performance measures in facilities that implemented a quality improvement project, engagement with "clinical champions" and an ICU-Family Meeting template | Cohort study | Separate medical/cardiac ICU, surgical, and mixed medical-surgical | Not reported | Participating facilities had teams comprised of "clinical champions" from palliative medicine and ICU services | Not specified |
| Anderson A, et al. 1991 | United States | Development and implementation of a bereavement program in the Medical Respiratory intensive care unit. | Qualitative | Medical Respiratory ICU | Follow-up completed for 38 patients; 47% completed the evaluation form | N/A | Nurses in the Medical Respiratory ICU |
| Azad MA, et al. 2020 | Canada | To explore the influence of personalized paintings created to honor deceased critically ill patients on family members’ bereavement experience | Qualitative descriptive analysis. | 21-bed medical-surgical, university-affiliated tertiary ICU | 22 family members | N/A | Artist (M.A.A.) who is an internal medicine resident with 15 years of painting experience. |
| Barnato AE, et al. 2017 | United States | To assess the feasibility, acceptability, and tolerability of storytelling among  bereaved surrogates involved in a decision to limit life support in the ICU. | Pilot single-blind trial | 5 ICUs (trauma, cardiovascular, 2 medical, and 1 mixed medical-surgical) | 32 bereaved surrogates | N/A | Trained interventionist |
| Bazzano, et al. 2024 | Italy | To investigate the role of ICU diaries in the grief process of family members of deceased ICU patients | Qualitative | General ICU | Nine family members of seven deceased ICU patients | ICU nurses and team members | ICU staff (handover of diaries) |
| Beiermann M, et al. 2017 | United States | The primary aim was to study the bereavement experience for families in the ICU; secondary aim was to measure nurses' perception of end-of-life care, and a third was to evaluate the impact of the ECG Memento^©^ by families and nurses. | Prospective, descriptive study design, with a postsurvey methodology | ICU and intermediate cardiac care unit | 28 family members  38 nurses | Research study nurse | Not specified |
| Brekelmans ACM, et al. 2022 | Netherlands | The aim of the study was to evaluate the quality of ICU ‘end-of-life care’ as well as the current bereavement support strategies in a large tertiary hospital, reported by bereaved family members of patients who were admitted to ICU who received bereavement support. | Cross-sectional single site study | Not specified | 95 relatives of deceased ICU patients | N/A | Specialized ICU nurse conducts the initial phone call, and a member of the research team approached relatives of deceased ICU patients by phone to participate in this study. |
| Combe D. 2005 | UK | 1) The purpose of our intensive-care follow-up clinic was twofold: firstly, to help patients after ICU with a variety of potential problems and secondly, to seek their perceptions of their experience in ICU, with a view of using this information to improve care 2) Evaluate feedback to the introduction of retrospective diaries of stay at the follow-up clinic 3) Evaluate implementation of prospective diaries | Pilot study | Not specified | 28 families | Anyone who had been involved in the care of the patient was invited to write in the diary, as well as loved ones and relatives. | ‘Follow-up sister’ |
| Dekeyser, et al. 2021 | France | To evaluate the impact of a psychologist-physician post-death meeting (PDM) as part of a bereavement program | Pilot observational study | Mixed ICU in a non-academic hospital | 53 bereaved relatives; 12 (23%) attended the PDM | N/A | Post-death meeting outside ICU with ICU psychologist and physician |
| Erikson A, et al. 2019 | United States | The study aim was to describe cardiac ICU families' opinions of six common components of a bereavement program: bereavement brochure, sympathy card, follow-up phone call, memory box (e.g. lock of hair, fingerprint), counselling and memorial service. In this cardiac ICU, a bereavement brochure and sympathy card were already provided, but the other potential components had not been implemented. Therefore, only results related to the sympathy card and bereavement program are included in here. | Qualitative Descriptive | Cardiac ICU | 12 family members | Not specified | Cardiac ICU staff |
| Hall B, et al. 1994 | UK | Determine how to improve nurses' service and to provide (if possible) a follow up service appropriate to their needs by asking bereaved relatives in the intensive therapy unit (ITU) what they would have liked during their time in the unit and afterwards, during their bereavement. | Qualitative | 3-4 bed Intensive Therapy Unit | 16 recently bereaved relatives | N/A | Nurse |
| Harris D, et al. 2021 | UK | To evaluate the service delivery of the bereavement care that is provided on a 20-bed general ICU. In addition, the study aimed to explore the experience of bereavement care currently provided in the ICU and to compare the findings with practice nationally and internationally | Qualitative | Medical/Surgical/Transplant ICU | 31 nurses  1 allied health professional  3 members of the medical team | N/A | Not specified (although may have included RN, charge nurse, healthcare assistant, doctor, allied health professional). |
| Johansson M, et al. 2018 | Sweden | To explore how family members experienced the use of a diary when a relative does not survive the stay in the intensive care unit (ICU). | Qualitative | Not stated | 9 family members (relatives and friends who visited the patient) | Family members | First author (also works as ICU nurse) |
| Kentish-Barnes N, et al. 2017 | France | Understand bereaved family members’ experience of receiving a letter of condolence. | Qualitative study (part of larger randomized, controlled, multicenter study) | 22 ICUs | 12 relatives from each of 22 participating centers | N/A | Patient's physician and nurse wrote the condolence letters. Psychologist, sociologist, and a research nurse conducted follow up phone calls. |
| Kentish-Barnes N, et al. 2017 | France | To test the hypothesis that a condolence letter, compared to no condolence letter, alleviated grief symptoms in relatives of patients who had died in the ICU | RCT | Not stated (although mentioned that they had "considerable expertise in end-of-life care") | 242 relatives of patients who died in 22 ICUs (123 in intervention, 119 in control) | N/A | Physician and nurse |
| Kentish-Barnes N, et al. 2022 | France | Evaluate whether a proactive communication and support intervention would improve relatives’ outcomes of patients dying in the ICU | RCT | 34 ICUs (medical and surgical) | 875 relatives of ICU patients recruited (484 in intervention, 391 in control). 379 and 309 participants completed follow-up. | Physician and patient’s primary nurse | Physician and nurse |
| Kock et al. 2014 | Sweden | To evaluate family perceptions of follow-up meetings post-ICU death | Quality improvement evaluation via retrospective questionnaire | ICU at Sahlgrenska University Hospital/Östra | 84 family members of 56 deceased ICU patients; 46 attended follow-up meetings | N/A | Follow-up meeting 4–6 weeks post-death with ICU physician, nurse, social worker |
| Lichtenthal WG, et al. 2022 | United States | (1) to develop and refine EMPOWER (Enhancing and Mobilizing the POtential for Wellness and Resilience), a brief manualized cognitive- behavioral, acceptance-based intervention) using feedback from bereaved surrogates and from expert critical care and mental health clinicians and (2) to determine preliminary feasibility, acceptability, and effects of EMPOWER on surrogate mental health and patient outcomes | Part 1 used a modified Delphi approach that involved gathering opinions from experts, keeping feedback anonymous, identifying and summarizing feedback themes, and circulating modifications back to experts until consensus is reached (this included surrogates/bereaved loved ones). Part 2 was an open single-arm pilot trial of EMPOWER implemented in the ICU of an urban academic medical center. | ICU and step-down unit | Part 1: 5 bereaved surrogates, 10 healthcare professionals  Part 2: 10 surrogates | Licensed clinical psychologist, master’s level advanced clinical psychology doctoral student, an experienced licensed social worker, and a more recently licensed social worker | Assumed to be the same as the in-hospital component |
| McAdam JL & Puntillo K. 2018 | United States | 1) To evaluate the effectiveness of bereavement follow-up on family members’ anxiety, depression, posttraumatic stress, prolonged grief, and satisfaction with care; 2) to evaluate the use, need, and desirability of bereavement services offered to families who received bereavement follow-up and those who didn't. | Pilot study with prospective cross-sectional design | Medical-surgical ICU and Cardiac ICU | 40 family members (30 in intervention, 10 in control) | N/A | A member of the Bereavement Team |
| Melby AC, et al. 2020. | Norway | Increase knowledge regarding the experiences of bereaved family members who received the intensive care diary kept by registered nurses for them of a loved one after death. | Qualitative with phenomenological approach | Not stated | 6 bereaved family members (5 participated in interview, 1 corresponded via email) | ICU staff nurses | First-author (ICU nurse) |
| Mosenthal AC, et al. 2008. | United States | Determine if early, structured communication via an interdisciplinary model for palliative care integrated into the trauma and surgical ICU would improve end-of-life care practice. | Prospective, observational study | 14 bed trauma-surgical ICU | Not reported | Physician, nurse, counselors, pastor | N/A |
| Neville TH, et al. 2020 | Canada and United States | To characterize and enumerate the keepsakes that were created as part of the 3WP and to understand their value from the perspective of bereaved family members. | Qualitative analysis | Not specified? | 75 family members were interviewed, data gathered from 60 transcripts | Clinicians | N/A |
| Neville, et al. 2023 | Canada and United States | To assess whether the 3 Wishes Program is associated with improved family ratings of end-of-life (EOL) care | Observational quality improvement study with survey-based evaluation | 6 adult ICUs in a 2-hospital academic healthcare system (UCLA) | Deceased ICU patients and their bereaved family members; N = 314 survey respondents; 117 patients received the intervention | Wishes fulfilled at bedside by ICU clinicians and staff with input from families | N/A |
| Pinnington & Westwood. 2024 | UK | To describe the implementation and impact of a one-minute pause at the time of death in ICU | Descriptive report/quality improvement | Southport Critical Care Unit, UK | Not specified; anecdotal reports from staff and families | ICU bedside staff and interprofessional team | N/A |
| Platt. 2004 | UK | Evaluate the incorporation of a memorial service in the critical care unit and determine if it met the needs of those attending the service | Qualitative study (audit) | Critical Care Unit | 320 bereaved family members/friends attended service, 52 questionnaires distributed, 33 returned | N/A | Nursing staff, Head of Pastoral Care |
| Poppe C, et al. 2019 | Belgium | To evaluate the communication and emotional support provided to the donor families in the Belgian ICU's and ED's to improve common procedures. | Qualitative (utilized a participatory approach and focus groups) | ICU and emergency department | 203 questionnaires distributed to families, 64 respondents | N/A | Transplant coordinators distributed questionnaire |
| Renckens, et al. 2024 | Netherlands | To examine experiences with and needs for aftercare among bereaved ICU relatives before and during the COVID-19 pandemic | Mixed-methods study (quantitative survey + qualitative interviews) | Six ICUs | 90 survey respondents and 20 interviewees who had lost a loved one in the ICU | N/A | Follow-up conversation post-death with ICU physician/nurse |
| Riegel, et al. 2023 | Australia | To explore families’ experiences of memory making in ICU and its use in early bereavement | Descriptive qualitative study | 17-bed tertiary ICU/high-dependency unit | 21 family members of deceased patients | Memory making items prepared at bedside with ICU nurses/support from staff | N/A |
| Ross MW. 2008 | United States | Implement a single-center bereavement program | Qualitative | Cardiac ICU | >160 family member responses to questionnaire | N/A | Two nurses (including author) |
| Santiago C, et al. 2017 | Canada | 1) Develop and administer an inter-professional, multi-component bereavement program for family members of patients who died in the ICU consisting of a bereavement brochure, sympathy card, telephone follow up, and an invitation to attend a memorial service; 2) measure the feasibility of implementing each of the program components; and 3) determine family member attitudes towards the program components and overall satisfaction. | Pilot, observational study | Medical-surgical ICU | 30 family members | A chaplain (or bedside nurse if chaplain was not available) | Sympathy card mailed by study team, telephone follow-up call conducted by ICU social workers, memorial service invitation developed and delivered by the Spiritual Care Department |
| Schenker Y, et al. 2015 | United States | 1) Describe a conceptual framework underlying the beneficial  mental health effects of storytelling and 2) Present formative work developing a storytelling  intervention to reduce distress for recently bereaved surrogates. | Case series | Medical ICU | 6 surrogate family members | N/A | Facilitators part of research team |
| Schoeman T, et al. 2018 | Australia | 1) To determine if relatives of an Australian critically ill population were interested in using ICU diaries and 2) to determine the prevalence and impact of ICU diaries upon symptoms of PTSD, depression, and anxiety in relatives of an Australian critically ill population. | Prospective, observational, exploratory study | Mixed (medical/surgical) ICU | 60 family members (36 had “completed” diary at follow-up) | Study investigator (physician) | Study investigator (physician) |
| Showler, et al. 2022 | Australia | To evaluate the impact of bereavement follow-up (phone call or condolence letter) on psychological outcomes among ICU bereaved relatives | RCT | Single-centre tertiary ICU (Royal Melbourne Hospital) | 71 bereaved family members of ICU decedents; randomized to condolence letter, phone call, or no contact | N/A | Phone call and condolence letter post-discharge by ICU physician and trained support team |
| Takaoka A, et al. 2020 | Canada and United States | To explore family member and clinician experiences with receiving or sending handwritten sympathy cards upon the death of patients involved in a personalized end-of-life intervention, the 3 Wishes Project. | Qualitative analysis | ICU | Interviews and focus groups held with 171 family members and 222 clinicians at four centers. Sympathy cards were discussed during 32 interviews (by 25 family members and 11 clinicians) and 2 focus groups (8 clinicians). | Interprofessional clinicians | Interprofessional clinicians |
| Takaoka A, et al. 2021 | Canada | To explore the interface between the 3WP and organ donation as experienced by families, clinicians, and organ donation coordinators. | Qualitative | x4 ICUs - type not specified | 18 family members,17 clinicians, and 6 organ donation coordinators | Bedside clinicians | Study investigators |
| Whitmer M, et al. 2007 | United States | Share a nursing-led innovation consisting of the creation, implementation, use, and evaluation of a grieving cart at Banner Good Samaritan Medical Center, and the bereavement support process in place at the hospital. | Qualitative | Medical-Surgical ICU, Trauma ICU | 65 families received follow-up phone call | Led by bedside RN, also included nursing assistants, health unit secretaries, unit chaplain, palliative care NP, and music therapist | Palliative Care NP |
| Williams R, et al. 2003 | UK | Present a bereavement after-care service, the experiences of those involved in it, and the feedback received for it from bereaved family members. To increase awareness of the issues surrounding death and dying with critical care. • To provide a support network for patient’s relatives and carers • To provide a support network for the multidisciplinary team. • To develop guidelines for practice when caring for dying patients and their relatives. • To increase awareness of patient’s religious and cultural beliefs concerning death and dying. • To evaluate the effectiveness of the service 1 year on. | Qualitative | General ICU, Cardiac ICU | First, second, and third meetings had 8,12, and 8 families attend, respectively | Nurse, senior doctor | Members of bereavement support group (hospital chaplain, nurses, healthcare assistants, physiotherapists) |
| Yeager S, et al. 2010 | United States | Provide an overview of the specific items that made up the Embrace Hope intervention (a plan of care to help patients and. their families through the dying process), and apply the intervention to a specific patient case | Qualitative | Neurocritical Care Unit | 74 surrogates (38 completed questionnaire prior to intervention, 36 after) + 1 case study | Attending physician, nurse practitioner, pastoral care representative, communication liaison. | Unit clerk/communication liaison, hospital staff |
| Yeo. 2021 | Australia | Evaluate an initiative (in an Australian setting through a novel collaboration between DonateLife South Australia (DLSA) and the ICU of the Royal Adelaide Hospital) aimed at ensuring consideration of organ and tissue donation at the end of life and providing structured bereavement follow-up for families of patients who die in the ICU. | A Plan-Do-Study-Act method of quality improvement | Quaternary ICU (general adult medical and surgical caseload) - major referral centre for trauma, neurosurgery, cardiac surgery, haematology, and oncology patients | 124 of 201 (62%) family members completed telephone follow-up | Bedside nurse | Five experienced clinicians (medical and nursing) |

Table 2: Bereavement Interventions (Brief)

| **Intervention** | **Studies** | **Characteristics/ features of intervention** | **Overall Assessment of the Intervention** (positive, neutral, or negative) |
| --- | --- | --- | --- |
| Memorial service | [1–4] | Spiritual Care, chaplain or nurse led. May include a flower ceremony, reading of poetry/prose, invitation to light candle in hospital chapel, refreshments, memorial book containing names of deceased. | Positive[1–4] |
| Condolence letter/ sympathy card | [3,5–14] | Pre-printed/generic or personalized/hand-written.  Sent between 1 week to 1 year following death. | Positive[3,5,6,9–13]  Neutral[7,14]  Negative[8] |
| Mementos | [1,12,15–18] | Examples: Personalized painting, ECG Mementos©, printed word clouds, family photographs, fingerprints, handprints, locks of hair, two-part key rings, forget-me-knot seeds, teddy bears | Positive[1,12,15–18] |
| Diary (completed by staff or family) | [19–23] | Completed by ICU staff, family, and/or patient.  May include photos. | Positive[20,23]  Neutral[19,21,22] |
| Storytelling | [24,25] | Non-judgmental elicitation of the story (antecedents to patient’s ICU admission, the ICU experience and decision process, and aftermath of the patient’s death) | Positive[24,25] |
| Personal/ individualized final wishes | [4,12,17,26–28] | Honoring patient/family final wishes (e.g. nonhospital blanket, decorating room to reflect identity, playing favorite music, infusing calming scents in room, facilitating holiday celebrations/spiritual ceremonies, supporting difficult conversations, sunset photo on door to alert staff to additional supports needed, quiet space for families) | Positive[4,12,17,26–28] |
| Booklets/referral to resources | [1,3,4,6,9,10,12,13,27,29] | Brochures, booklets, information for patients and families. May contain information on grief, grief support, funeral planning, financial arrangements, obtaining death certificates.  Provided by chaplain or bedside nurse. | Positive[1,4,6,9,10,12,13,27,29]  Neutral[3] |
| Meeting with ICU physician and/or nurse/and/or other | [4,27,29–35] | Family conference, an ICU-room visit, meeting after the patient’s death, interdisciplinary family meeting.   - May include physicians, nurses, families, palliative care NP, chaplain. - May be within 72 hours of admission, or to prepare the relatives for the imminent death, to provide active support, to offer condolences and closure. - May be routine or upon request. | Positive[4,27,29–32,34]  Neutral[33] |
| Follow-up phone call | [3,5,9,10,13,14,36,37] | Timeframe: 2 weeks to 3 years, most commonly within 6 months.  Performed by nurse (specialized, bedside, or research), psychologist, sociologist, social worker,  To assess how bereaved loved ones are doing, offer sympathy, identify additional needs. | Positive[3,5,9,10,13]  Neutral[14,36,37] |
| Training for staff | [12,29] | Formats: interactive webinars, interdisciplinary role-play, coaching, educational sessions.  Content: key communication strategies and skills, cultural considerations of dying, institutional resources, symptom onset/ management.  Target: patient care technicians, nursing staff, physicians. | Positive[12,29] |
| Other | [27,38,39] | Six discrete ∼15-min modules to reduce experiential avoidance, grief, anxiety, and peritraumatic distress.  Two booster calls, acute meaning-centered bereavement support  Grieving cart with refreshments, English and Spanish versions of the Bible, Koran, and Book of Mormon, and pamphlets about grief and bereavement.  One-minute silence observed with staff and families immediately post-death, using set words and symbols | Positive[27,38,39] |

# Table 3: Summary of Findings Mapped to Core Outcome Set

| **Core Outcomes/Dimensions** | **Assessed By** | **Studies Reporting Outcome** |
| --- | --- | --- |
| *Ability to Cope with Grief* |  |  |
| Negative and overwhelming grief | DRS, ICG; follow-up phone call; informal meeting; ICG; PG-13; PG-13; PG-13; BFS | [8,9,19,24,28,30,36,38,39] |
| Communication and connectedness | Simple evaluation form; semi-structured interview; storytelling; interview; follow-up phone call, unsolicited feedback; interview; questionnaire; questionnaire; follow-up phone call, questionnaire; storytelling; semi-structured interview; focus group, interview; follow-up phone call; survey; semi-structured interview; BFS | [2,3,5,7,10–13,15,20,21,24–28,35] |
| Understanding, accepting and finding meaning in grief | Semi-structured interview; ICG; informal meeting; healthcare-provider questionnaire; interview; follow-up phone call, unsolicited feedback; interview; semi-structured interview/content analysis; questionnaire; storytelling; focus group, interview | [1,7,10,15,17–21,23–26,34,35] |
| Finding balance between grief and life going forwards | Semi-structured interview; interview; questionnaire; questionnaire | [2,10,15,21,23] |
| Accessing appropriate support | Self-reported mental health/grief support utilization; semi-structured interview; healthcare-provider questionnaire; Donor Family Questionnaire; attendance at support meetings; semi-structured interview | [1,4,13,24,32,34,37] |
| *Quality of Life and Mental Wellbeing* |  |  |
| Participation in work and/or other regular activities | Questionnaire | N/A |
| Relationships and social functioning | Semi-structured interview; healthcare-provider questionnaire | [15] |
| Positive mental wellbeing | Semi-structured interview | [15] |
| Negative mental and emotional state | IES-R, HADS, PRIME-MD-PHQ, SUDS, PICS-F; HADS, IES-R; HADS, IES-R; HADS, IES-R, GAD-7, PHQ-9; HADS, IES-R; storytelling, SUDS; IES-R, DASS-21 | [8,9,14,22,24,25,30,33,38] |

Impact of Events Scale-Revised (IES-R), Hospital Anxiety and Depression Scale (HADS), Primary Care Evaluation of Mental Disorders Patient Health Questionnaire (PRIME-MD-PHQ), Decision Regret Scale (DRS), Inventory of Complicated Grief (ICG), subjective units of distress (SUDS), Post-Intensive Care Syndrome – Family (PICS-F), Prolonged Grief 13-item questionnaire (PG-13), Patient Health Questionnaire (PHQ-9), Generalized Anxiety Disorder-7 (GAD-7), Traumatic Grief Inventory-Self Report (TGI-SR), Bereaved Family Survey (BFS)

**Studies that did not map findings to core outcomes:**

[6,16,29,31]

eSupplement: Bereavement Interventions (Full)

| **Intervention** | **Studies (by #)** e.g. 1, 3, 5... | **Characteristics/ features of intervention**  e.g. differences in timing, characteristics, etc. | **Findings**  What were the outcomes? (i.e. evidence for/against the intervention & it’s implementation.) | **Authors’ (Verbatim) Conclusions** | **Overall Assessment of the Intervention** (positive, neutral, or negative) |
| --- | --- | --- | --- | --- | --- |
| Memorial service | [1] | Along with information booklet and bereavement box, includes invitation to a memorial service a few months after the event | A total of eight respondents reported receiving positive feedback from families | Participants described the different interventions they used as part of providing bereavement care, notably making memories. The bereavement box received high praise and all three critical care areas of the hospital have now implemented the same bereavement box, offering equal access to bereavement resources. | Positive |
|  | [2] | Memorial service, which involved flower ceremony, reading of poetry/prose, and followed by invitation to light candle in hospital chapel. | Number/percentage of positive responses to aspects of memorial service: venue suitable - 32/97%, poetry-prose helpful - 31/94%, right religious input - 17/52%, involvement of staff adequate - 27/82%, formality of service right - 20/61%, happy with no singing - 18/55%, happy to not contribute - 18/55%, like sitting round a table - 33/100%, flower ceremony appropriate 31/94%, glad attended - 32/97%, like to be re-invited 24/72% | In carrying out our audit, we were seeking to evaluate the effectiveness of our service and to further reinforce a need to continue with it each year. The evidence gained from the audit supports the need to continue with the service as an annual event, and that currently only minor changes need to be made in the planning, organisation and delivery of the service. | Positive |
|  | [3] | Invitation to attend a memorial service (developed and delivered by the Spiritual Care Department). Service held quarterly. | Only 3 participants responded to the question about whether they think they would attend the memorial service: one replied yes, one replied no, and one did not remember. No one responded to the question asking about the helpfulness of memorial service. Overall, on a scale of 0–10, the mean rating for program helpfulness was 7 (range 5–10), with 44% rating the program 7 or higher. | Our study shows that a formal, multi-component bereavement program for family members of patients who die in the ICU is feasible to implement, yet the value of each individual component of the program is family member dependent. The development of bereavement follow-up programs should take into consideration the resources available in the institution and the unique needs of individual families for optimal impact. | Positive |
|  | [4] | Held in education centre (away from clinical environment) q6 months. Hospital chaplain makes opening speech, and relatives sit in small groups with member of support group and talk about experiences. Activity such as planting bulbs is offered to those relatives who wish to take something away from the day. During this time, there is a memorial book, containing the names and dates of all patients who have died within the ICU. The meetings run for 3–4h, refreshments and light lunch provided. Relatives can visit the ITU units if desired, with support of the bereavement group. | During audit period, 235 deaths, three relative support meetings. The majority of comments made by relatives were positive. | The feedback we have received from relatives, nursing and medical staff suggests that the service we are providing is meeting many of the bereaved relatives needs. However, more structured evaluation and research is planned for the future to evaluate the service and what relatives want from it. | Positive |
| Condolence letter/ sympathy card | [5] | Sympathy card sent @ 1-2 weeks, +/- @ 3-6 months, and handwritten note @ 1 year | Helpful by all who responded/ completed evaluation. Additional comments from families noted to be positive. | Our Bereavement Follow-up program has been viewed as a success by both staff and families. We have shown how a group of staff nurses, given support and some minimal time away from direct patient care responsibilities, can develop and implement a program of follow-up after patient deaths. | Positive |
|  | [6] | Pre-printed sympathy card hand signed by cardiac ICU staff, mailed @ 2 weeks after death. | Family used words such as "meaningful" and "heartwarming" to describe card, reported timing as appropriate, preferred card signed by staff who cared for loved one, overall rated as neutral in providing comfort. | Participants varied in their opinions about what bereavement services may be useful; however, this study provides insight about services that cardiac critical care units may consider when adding bereavement support. | Positive |
|  | [7] | Personalized condolence letter written by nurse and physician | Benefits of receiving a letter of condolence include humanizes the medical institution (feeling of support, confirmation of the role played by the relative, supplemental information). However, also common ambivalence about the letter of condolence’s benefit. | This study puts forward the benefits of receiving a letter of condolence but also shows that relatives sometimes experience doubts and ambivalent feelings about the letter, such as pain, suspicion, and a social obligation to answer the letter. Although the study shows global satisfaction, it also shows that the letter is not systematically beneficial, and healthcare workers must strive to adapt bereavement follow-up to each individual situation. | Neutral |
|  | [8] | Condolence letter prepared within 3 days of patient's death and sent by standard mail 15 days after (recognized death and name of patient, mentioned personal impression, recognized the family member, offered help, and expressed sympathy). | No significant difference in outcomes at 1 month, after 6 months HADS score, depression subscale, and depression symptoms significantly worse in intervention group. Similar prevalence of complicated grief symptoms | In conclusion, in our trial, a condolence letter did not alleviate grief symptoms in relatives of patients who died in the ICU. Unexpectedly, the intervention was associated with higher prevalences of symptoms of depression and PTSD. Our findings do not support the sending of a condolence letter to bereaved relatives as the sole, routine post-ICU intervention. | Negative |
|  | [9] | One week after death, family received preprinted condolence card hand-signed by staff who knew patient. At 1 year sent a handwritten note of condolence. On what would have been patient’s birthday, card sent reiterating ICU’s continued support and remembrance of pt. | No sig. difference in depression, anxiety, PTSD, and ICU satisfaction scores. Significantly more family members in non-bereavement group experienced prolonged grief (3 vs 0 participants). Most found the condolence card to be meaningful/very meaningful. 47%. preferred that f/u only occur for 1 month after death. | Bereavement follow-up after an ICU death helped lower the risk of prolonged grief and may help lower the risk of PTSD in family members. It did not affect anxiety, depression, or satisfaction with care. The results regarding the most useful components of the bereavement program were mixed, but it was clear that ICU families do want this type of support. | Positive |
|  | [10] | Sent a sympathy card @ 2 weeks and mailed a handwritten note at both 6 months and 1 year. | 158/160 responses expressing positive reactions to the bereavement program.  “Touched that we remembered them and their loved one and really appreciated the handwritten letters. Many said that few people keep in touch after the funeral— people seem to think the families need to get on with their lives and get over the death of their loved one. They appreciate our acknowledgement of the fact that grief has no timetable”. | Throughout the program, bereaved family members have responded positively, and the nursing staff in our busy unit continues to keep in touch with them. Our bereavement program is providing comfort for the families and closure for the nurses. | Positive |
|  | [3] | A sympathy card was mailed to next of kin 10 days after the patient's death by the study team | All respondents (n=11) reported receiving the sympathy card and finding it meaningful. When asked about how they felt receiving the card, the respondents described feeling “touched that someone took the time”, “thankful because (they) were not forgotten”, “surprised”, “relieved”, “happy and sad”, and “cared for”. Overall, on a scale of 0–10, the mean rating for program helpfulness was 7 (range 5–10), with 44% rating the program 7 or higher. | Our study shows that a formal, multi-component bereavement program for family members of patients who die in the ICU is feasible to implement, yet the value of each individual component of the program is family member dependent. The development of bereavement follow-up programs should take into consideration the resources available in the institution and the unique needs of individual families for optimal impact. | Positive |
|  | [11] | Sympathy cards were mailed to families between 2-8 weeks after the patient's death. Sympathy cards included a few personal words to bereaved family members that were handwritten by clinicians. Cards were custom designed with a brief condolence message printed inside. | Family members experienced sympathy cards as tangible expression of continued connection beyond the patient’s illness. Reflected in 3 themes: cards represent a valued demonstration of shared humanity; personalized, authentic messages from several clinicians are features that render cards meaningful to those sending and those receiving them; and sympathy cards extend compassionate patient- and family-centered end-of-life care during bereavement. Example of feedback from one family member: “[They] were very specific, loving notes. I mean, that card was worth gold.” | Writing personalized sympathy cards to family members of deceased patients can be perceived as a meaningful, compassionate gesture and represents a simple example of tailored bereavement care. The content included in a sympathy card and the context in which it is written could influence the recipient’s experience. Key features are the specificity of the messages and the sincerity of the sentiments. In today’s digital era, handwritten, individualized, unstructured messages represent heartfelt, valued expressions of condolence for families and clinicians alike. | Positive |
|  | [12] | Unit sympathy card with seed packet (packet of wildflower seeds encased in a dark blue velvet bag) attached to the violet tab in the blue chart for signatures, and after the patient discharges kept in metal bin by UC desk and mailed to family by UC or communication liaison 5 days after patient discharged from unit. | Statistically significant changes in the perceived emotional support and overall care provided were noted on follow-up surveys. Followed one participant - noted appreciation for care at end of process several weeks later in letter to the unit. | Through hard work and emphasis on supporting patients and their families at the end of life, quality care can be provided within the walls of inpatient critical care units. Emphasizing the skill sets of an entire multidisciplinary team enables a structured intervention that optimizes caring while enabling a manageable workflow for team members. | Positive |
|  | [14] | Letter personalized with names, included contact details and resources; sent 4 weeks post-death | No statistically significant effect on anxiety, depression, PTSD, or complicated grief vs. no contact | Condolence letters did not meaningfully alleviate psychological distress at 6 months | Neutral |
|  | [13] | After death family member given a bereavement pack, which included a generic condolence letter. | Likert scores (ranging from 1 (least satisfied) to 5 (most satisfied)) were consistently high (median >4). | The focus on quality of end-of-life care promoted recruitment and potentially identified previously missed possible donors. Bereavement telephone follow-up harnessed the insights of consumers to identify opportunities to improve end of-life care. The feasibility of this collaborative strategy should be further tested in other healthcare environments. | Positive |
| Mementos | [15] | Personalized painting | Overarching theme: Art to facilitate healing. Domains: 1) Cocreation 2) painting narratives 3) postmortem connection 4) legacy | This qualitative study’s findings suggest that the creation of personalized paintings commemorating the lives of patients may help foster legacy and postmortem connections with clinicians and may help family members in their healing process. | Positive |
|  | [16] | Laminated ECG Mementos© (up to 8 for larger families, if requested) | 61% found it extremely/very helpful, 41% viewed it daily 6 to 8 weeks after death. 25% found it somewhat or only slightly helpful, and 14% never looked at it/found it helpful. | Most families responded positively to this novel bereavement tool, which may aid families as they transition from anticipatory grieving to bereaved status. Further studies are needed to evaluate the ECG Memento as an innovation on a wider scale and to develop additional interventions to positively impact the grieving process for families. | Positive |
|  | [1] | Bereavement box – includes fingerprints, handprints (which can be made into jewellery), locks of hair, two-part key rings, forget-me-knot seeds and electric candles (for ambience) | A total of eight respondents reported receiving positive feedback from families | Participants described the different interventions they used as part of providing bereavement care, notably making memories. The bereavement box received high praise and all three critical care areas of the hospital have now implemented the same bereavement box, offering equal access to bereavement resources. | Positive |
|  | [17] | Clinicians ask how they might bring comfort to pt/family in final hours/days, including creating keepsakes for family to take home. Included printed word clouds, family photographs, thumbprints, locks of hair). Qualitative analysis revealed two major themes regarding keepsakes at the EOL: 1. keepsakes are tangible items that are highly valued by family members and 2. the creation of the keepsake with the clinical staff is a valuable experience and viewed as a gesture of compassion. - - Overall, both the offering to create the keepsake and the final products themselves are perceived by family members as being helpful. | Qualitative analysis revealed two major themes regarding keepsakes at the EOL: 1. keepsakes are tangible items that are highly valued by family members and 2. The creation of the keepsake with the clinical staff is a valuable experience and viewed as a gesture of compassion. Overall, both the offering to create the keepsake and the final products themselves are perceived by family members as being helpful. | In summary, keepsakes are common wishes that frontline clinicians in the ICU are able to provide in our multicenter evaluation of the 3WP. Both the offering to create the keepsake and the final products themselves are perceived by family members as being helpful. | Positive |
|  | [18] | Memory making items offered during end-of-life care, selected by families and created by ICU staff | Items helped relatives access memories, assign meaning, and preserve connections during early grief | Tangible objects assisted recipients during grief work in early bereavement; timing and family choice are important | Positive |
|  | [12] | Embrace Hope cloth envelope includes “Hope in Remembering” poem, hand-tracing card, and “A Lock of Love” packet. Explanation of hand tracing and/or “A Lock of Love” provided to family and verbal consent documented on flow sheets as appropriate. Obtain snippet of hair/hand tracing for family with as little or as much family participation as they desire. | Perceived emotional support and overall care provided were noted on follow-up surveys. Followed one participant - noted appreciation for care at end of process several weeks later in letter to the unit | Through hard work and emphasis on supporting patients and their families at the end of life, quality care can be provided within the walls of inpatient critical care units. Emphasizing the skill sets of an entire multidisciplinary team enables a structured intervention that optimizes caring while enabling a manageable workflow for team members. | Positive |
| Diary (completed by staff or family) | [19] | Diary kept in bright folder at bedside and began with brief summary of events leading up to admission. Relatives encouraged to read/contribute. Diaries collated/bound, given to either patient or bereaved loved one. | Bereaved families stated they were pleased to receive diary/glad to have something to look back on, and concrete memory of their loved ones’ last days. Minority who reported feeling upset after seeing photographs of their loved ones after they had died. | This pilot study has established the logistics of compiling ICU diaries and their general acceptability to patients. The feedback has been very positive from patients, relatives and staff who have experienced them. | Neutral |
|  | [20] | Completed by family | Three themes (diary promoted rational understanding, emotional understanding, and social interactions) and six subthemes (the diary provided information, reflected the patient's everyday activities, reflected emotions, provided comfort, maintained communication, and maintained a relationship). Author’s Conclusion: Family members of nonsurvivors had a need to have the ICU time explained and expressed. The diary might work as a form of 'survival kit' to gain coherence and understanding; to meet their needs during the hospital stay; and, finally, to act as a bereavement support by processing the death of the patient. | Family members of nonsurvivors had a need to have the ICU time explained and expressed. The diary might work as a form of ‘survival kit’ to gain coherence and understanding; to meet their needs during the hospital stay; and, finally, to act as a bereavement support by processing the death of the patient. | Positive |
|  | [21] | Diary (including photos) for patients/ bereaved family members | Two main themes (suggesting mixed experiences) emerged from the analysis, which in combination suggested mixed experiences among the participants with using the diary in the grieving process: grateful to have received it, but also triggered powerful emotional reactions. 1. Receiving the diary - a mixed blessing. 2. Using the diary in the grieving process. | The diaries were used by the bereaved for comfort and support during their grieving process. The diary information helped impart a structure on the chaotic time that followed the death of their loved one. The diaries also helped them imbue their suffering with meaning and maintain a bond with the deceased. | Neutral |
|  | [23] | Narrative diaries written by ICU staff, returned to family post-mortem as part of follow-up program | Families described diaries as helping process grief, understand care, and maintain connection with the deceased | ICU diaries can help relatives understand what happened and support them in the grieving process | Positive |
|  | [22] | Written prospectively and addressed personally to individual pt. Began on day 3 with a summary detailing reason for admission. Involved daily documentation by bedside nurse. A final summary note completed by medical staff upon discharge from the ICU. Family members also given the opportunity to contribute to the diary. Then hand-delivered to the patient by the investigator. 20% of patients did not survive. | 36/60 family members (60%) had “completed” ICU diaries at follow up. Twenty-four (40%) had diaries which were missing entries on certain days and hence deemed ‘incomplete’. There was no association between PTSD at follow-up and percentage of useful completion of the diary. Irrespective of diary completion there was also no association between PTSD at follow- up and diary use. ICU diaries were often not completed and completion did not appear to be related to the incidence of stress, anxiety, depression and PTSD symptoms. Greater diary use (i.e. diaries considered complete) across the test period in populations with higher IES-R and depression scores | ICU diaries were often incomplete. No relationship was found between completion of diaries and improvement in symptoms of anxiety, depression or severe PTSD symptoms in relatives of patients in this index population. This may be because Australian families are generally not interested in maintaining a diary during a relative’s ICU stay. | Neutral |
| Storytelling | [24] | Non-judgmental elicitation of the story of the events leading up to the patient’s ICU admission, the ICU experience and decision process, and the aftermath of the patient’s death, w/ with empathic reflective statements and probes of interpersonal and intrapersonal content. | At 6-months 94% of storytelling subjects reported feeling “better” or “much better,” compared to 69% of control participants. | A clinical trial of storytelling among recently bereaved ICU surrogates is feasible, acceptable and tolerable. These subjects have a high symptom burden and report altruistic benefits from the opportunity to participate in research that might help others through this difficult experience in the future. | Positive |
|  | [25] | Storytelling intervention as opportunity to discuss participating in a decision to limit life-sustaining treatment for a loved one in ICU within 2-4 weeks of the patient's death After session, facilitator debriefed the subject regarding the experience of study participation, including questions about burdensomeness, acceptability, and perceived value. The initial semi-structured guide included questions to elicit three key domains of the story: the antecedents (the illness that brought the patient to the ICU), the ICU experience (including the decision to limit life sustaining treatment and the patient's death) and the aftermath (the surrogate's feelings or thoughts about the decedent, the ICU experience, and the decision to limit life-sustaining treatment). | All storytelling participants endorsed the intervention as acceptable, and 5 of 6 reported that it was helpful. Post-intervention SUDS ranged from 5-60 and were no higher than scores before the intervention. | In summary, we describe an innovative post-ICU intervention designed by a multidisciplinary team and refined through an open-label case series of recently bereaved surrogate decision makers. This work supports further evaluation of the safety and acceptability of the surrogate storytelling intervention in a phase II study and, ultimately, a larger randomized trial to assess efficacy. | Positive |
| Personal/ individualized final wishes | [17] | Clinicians ask how they might bring comfort to pt/family in final hours/days (providing pt with nonhospital blanket, decorating pt’s room with items that reflect identity, playing pt’s favorite music, infusing calming scents in room, facilitating holiday celebrations/spiritual ceremonies, and creating keepsakes for family to take home. | Qualitative analysis revealed two major themes regarding keepsakes at the EOL: 1. keepsakes are tangible items that are highly valued by family members and 2. The creation of the keepsake with the clinical staff is a valuable experience and viewed as a gesture of compassion. Overall, both the offering to create the keepsake and the final products themselves are perceived by family members as being helpful. | In summary, keepsakes are common wishes that frontline clinicians in the ICU are able to provide in our multicenter evaluation of the 3WP. Both the offering to create the keepsake and the final products themselves are perceived by family members as being helpful. | Positive |
|  | [26] | Participants identified multiple ways that the 3WP could offer support to families throughout the organ donation process, which we organized into 3 temporal phases: family decision-making (honoring patient’s wishes, supporting difficult conversations), preparing for donation (empowering families when they feel helpless, shifting focus to personhood), and postmortem family care (emphasizing fulfilled wishes when donation is unsuccessful, extending postmortem family care regardless of donation outcome.) Family follow-up in the 3WP may include sympathy cards, hospital memorial services, debriefing visits, grief counseling, and other connections, regardless of whether the patient is involved in organ donation. | The 3WP also facilitates reflection on other fulfilled wishes and positive end-of-life memories for families. Regarding the inability to implement donation after DCD, a donation coordinator shared how the 3WP could help by initiating acts of compassion to commemorate a patient.  Family follow-up in the 3WP may include sympathy cards, hospital memorial services, debriefing visits, grief counseling, and other connections, regardless of whether the patient is involved in organ donation.  Postmortem family support may be particularly helpful for consenting families of nondonors who would not otherwise receive as much organ donation coordinator follow-up. | As a personalized, semi-structured intervention, the 3WP encourages an individualized approach to end-of-life care, implementing final wishes for patients and their families that are adaptable to different settings and situations.  Our findings demonstrate how the 3WP and the option of organ donation present complementary opportunities for patients and families to engage in value-based conversations highlighting their own agency during the dying process. We identified the potential for each program to enhance the other. Mutual goals of the 3WP and organ donation that can be coordinated and strengthened include emphasizing personhood and bringing meaning to the death while honoring the patient’s legacy. The shared values of these 2 programs may help to incorporate organ donation and death into a person’s life narrative and incorporate new life into a person’s death narrative. | Positive |
|  | [27] | A sunset photo is placed on the patient's door (quiets the unit, other nurses support bedside nurse as they spend more time increasing familial support). Music therapists who have interacted with the patient in the past are also available to provide therapeutic support. | Families ‘‘felt very cared for" and that ‘‘the nurses understood how we were feeling" | The grieving cart has been an inexpensive, low-tech intervention that has resulted in a profound change in our ICU philosophy and the nursing care delivered to families just beginning the grieving process. The grieving cart serves as a symbol of caring and support for families and a way for nurses to complete the circle of caring in which aggressive, cure driven care becomes palliative care and peaceful death. | Positive |
|  | [4] | Special quiet room arranged for family members (nicely decorated, lockable room, with comfortable sofas) just outside of ICU. | The majority of comments made by relatives were positive. | The feedback we have received from relatives, nursing and medical staff suggests that the service we are providing is meeting many of the bereaved relatives needs. However, more structured evaluation and research is planned for the future to evaluate the service and what relatives want from it. | Positive |
|  | [28] | Implemented in 6 ICUs; wishes initiated near end-of-life by clinicians or project manager; supported by nurse champions. Included music, keepsakes, non-hospital blankets. | Higher scores for Emotional and Spiritual Support (7.5 vs 6.0, p=0.003); no significant difference for Respectful Care or BFS-Performance Measure | The 3WP is a low-cost intervention that may be a feasible strategy for improving the EOL experience | Positive |
|  | [12] | As families transition into grieving period, process begins in family conference room (closed room containing multiple chairs and couches, tissue boxes, water, phone access, and a wall quilt to create a private, comfortable setting to receive updates). Sign placed on patient room door (“please See Nurse Prior to Entering Room”) and magnet placed on white board identifying “Embrace Hope” | Perceived emotional support and overall care provided were noted on follow-up surveys. Followed one participant - noted appreciation for care at end of process several weeks later in letter to the unit | Through hard work and emphasis on supporting patients and their families at the end of life, quality care can be provided within the walls of inpatient critical care units. Emphasizing the skill sets of an entire multidisciplinary team enables a structured intervention that optimizes caring while enabling a manageable workflow for team members. | Positive |
| Booklets/referral to resources | [29] | Informational booklets for patients and families | Bereaved Family Survey-Performance Measure (BFS-PM) score improved | VA ICU-FM QI Initiative demonstrated an increase in documented FMs in VA medical center decedents, which is an important process measure of ICU care quality. | Positive |
|  | [6] | Bereavement Brochure - contained information on grief, funeral planning, financial arrangements, obtaining death certificates. | Allowed access to info on own time, can be reused, provided practical + emotional needs. Constructive feedback: timing issues - rushed, thrown at family; lack of services in the family's geographic area; information not relevant to some families | Participants varied in their opinions about what bereavement services may be useful; however, this study provides insight about services that cardiac critical care units may consider when adding bereavement support. | Positive |
|  | [1] | Information booklet on what to do after the death | A total of eight respondents reported receiving positive feedback from families | Participants described the different interventions they used as part of providing bereavement care, notably making memories. The bereavement box received high praise and all three critical care areas of the hospital have now implemented the same bereavement box, offering equal access to bereavement resources. | Positive |
|  | [9] | Upon pt’s death, family given bereavement brochure containing information on grief, funeral planning, financial arrangements, obtaining death certificates, and resources for grief support. One week after death, family received (along with condolence card) a practical tasks resource packet that reiterates info in bereavement brochure and contains info for settling deceased’s affairs and connecting with grief support groups. | No sig. difference in depression, anxiety, PTSD, and ICU satisfaction scores. Significantly more family members in non-bereavement group experienced prolonged grief (3 vs 0 participants).  Most remembered receiving the bereavement brochure/practical tasks packet, and most found these resources helpful. However, many did not use the resources. 47%. preferred that f/u only occur for 1 month after death | Bereavement follow-up after an ICU death helped lower the risk of prolonged grief and may help lower the risk of PTSD in family members. It did not affect anxiety, depression, or satisfaction with care. The results regarding the most useful components of the bereavement program were mixed, but it was clear that ICU families do want this type of support. | Positive |
|  | [10] | Provided a folder with list of support groups in New York, Connecticut, and Rhode Island, and 7 brochures addressing bereavement issues (@ approx 4 weeks). | 158/160 responses expressing positive reactions to the bereavement program.  “The booklets you sent were treasures. They dealt upfront with things that are so painful, like getting through birthdays, the holidays and that first anniversary ... What you are doing for the bereaved is incredible”. | Throughout the program, bereaved family members have responded positively, and the nursing staff in our busy unit continues to keep in touch with them. Our bereavement program is providing comfort for the families and closure for the nurses. | Positive |
|  | [3] | “After a Loved One Dies” brochure was provided to next of kin by a chaplain or bedside nurse. Pamphlet contains information about community support systems and educational materials about grief management. | Among survey respondents, 82% (n=9/11) replied that they had received the brochure and the majority (90%; n=9/10) reported reading it. Less than half (43%; n=3/7) found the brochure helpful while the remainder (57%; n=4/7) were neutral about its helpfulness. Overall, on a scale of 0–10, the mean rating for program helpfulness was 7 (range 5–10), with 44% rating the program 7 or higher. | Our study shows that a formal, multi-component bereavement program for family members of patients who die in the ICU is feasible to implement, yet the value of each individual component of the program is family member dependent. The development of bereavement follow-up programs should take into consideration the resources available in the institution and the unique needs of individual families for optimal impact. | Neutral |
|  | [27] | Information about the grief and bereavement process is given to families in the form of written materials. Pamphlets about grief and bereavement along with religious material (ex. bible) located in grieving cart. | Not commented on | The grieving cart has been an inexpensive, low-tech intervention that has resulted in a profound change in our ICU philosophy and the nursing care delivered to families just beginning the grieving process. The grieving cart serves as a symbol of caring and support for families and a way for nurses to complete the circle of caring in which aggressive, cure driven care becomes palliative care and peaceful death. | Positive |
|  | [4] | Once relatives given time to digest news, given hospital booklet ‘coping with bereavement’ along with leaflet informing them about support group. Contains practical information, contact details of people (registrars, funeral director). | The majority of comments made by relatives were positive. | The feedback we have received from relatives, nursing and medical staff suggests that the service we are providing is meeting many of the bereaved relatives needs. However, more structured evaluation and research is planned for the future to evaluate the service and what relatives want from it. | Positive |
|  | [12] | “What to Expect During This Transition” brochure given to family during grieving process. At time of death, after patient has been pronounced, Pastoral Care Bereavement packet added to Embrace Hope cloth envelope. | Perceived emotional support and overall care provided were noted on follow-up surveys. Followed one participant - noted appreciation for care at end of process several weeks later in letter to the unit | Through hard work and emphasis on supporting patients and their families at the end of life, quality care can be provided within the walls of inpatient critical care units. Emphasizing the skill sets of an entire multidisciplinary team enables a structured intervention that optimizes caring while enabling a manageable workflow for team members. | Positive |
|  | [13] | After death family member given a bereavement pack by the bedside nurse. The pack consisted of written information on loss and grief and practical information on arrangements commonly required after death in hospital, with links to useful bereavement resources. | Likert scores (ranging from 1 (least satisfied) to 5 (most satisfied)) were consistently high (median >4). | The focus on quality of end-of-life care promoted recruitment and potentially identified previously missed possible donors. Bereavement telephone follow-up harnessed the insights of consumers to identify opportunities to improve end of-life care. The feasibility of this collaborative strategy should be further tested in other healthcare environments. | Positive |
| Meeting with ICU physician and/or nurse/and/or other | [29] | Project aimed at improving ICU family meetings | 1 - Bereaved Family Survey-Performance Measure (BFS-PM) score improved | An ICU-FM QI Initiative demonstrated an increase in documented FMs in VA medical center decedents, which is an important process measure of ICU care quality. | Positive |
|  | [30] | In the intervention group, three meetings were held with relatives: a family conference to prepare the relatives for the imminent death, an ICU-room visit to provide active support, and a meeting after the patient’s death to offer condolences and closure. | The median PG-13 score at 6 months (primary outcome) significantly lower in the intervention group than control group, which also had fewer relatives with scores of 30 and higher. Among secondary outcomes, most variables studied were significantly better in the intervention than in the control group. At 1 month, relatives' experience, quality of death and dying as assessed by the relatives, and relatives' satisfaction all significantly better in intervention compared to control group, as were the IES-R scores and proportion of relatives whose scores indicated a high risk of PTSD at 3 and 6 months. | In conclusion, a three-step, physician-driven, nurse-aided support strategy decreased the prevalence of prolonged grief disorder among bereaved relatives. PTSD-related symptoms, as well as symptoms of anxiety, were less common in the intervention group than in the control group. The communication style used in the intervention deserves to be used widely in ICUs. | Positive |
|  | [31] | Program based on enhancement of communication between physicians, nurses and families around prognosis, pain/symptoms management, GOC, as well as early psychosocial support for families. Interdisciplinary family meeting within 72 hours of admission. | Bereavement/family support: 22% in the baseline group, and 6% in the intervention group. | Structured communication between physician and families resulted in earlier consensus around goals of care for dying trauma patients. Integration of early palliative care alongside aggressive trauma care can be accomplished without change in mortality and has the ability to change the culture of care in the trauma ICU | Positive |
|  | [32] | Evaluation whether provided communication and emotional support to family in context of organ donation met international recommendations (via Donor Family Questionnaire) | Families perceived physician as most supportive caregiver during most phases of the procedure: hospitalization phase (67.2%), conversations about the passing/brain death/ending therapy (59.4%), and aftercare (28.1%). In the parting process, nurses were more frequently endorsed as providing emotional support.  Information of the self-care group of donor families was appreciated by 58.6%. | The Donor Family questionnaire (DFQ) is a promising tool to enable caregivers to evaluate their approach of the families of donors in the future in a conform manner. We recommend hospitals to do this evaluation on a regular basis | Positive |
|  | [27] | The bedside nurse provides supportive care and information is provided about the dying process and how withdrawal of artificial life support happens. he unit chaplain and palliative care NP are alerted and are available for grief support. As the family shares food and drink, the nurse is able to spend time with the family answering questions, encouraging reflection about the patient’s life, and offering emotional support. The chaplain and palliative care NP also participate in the vigil. | Families ‘‘felt very cared for" and that ‘‘the nurses understood how we were feeling" | The grieving cart has been an inexpensive, low-tech intervention that has resulted in a profound change in our ICU philosophy and the nursing care delivered to families just beginning the grieving process. The grieving cart serves as a symbol of caring and support for families and a way for nurses to complete the circle of caring in which aggressive, cure driven care becomes palliative care and peaceful death. | Positive |
|  | [33] | Offered to bereaved families 3–4 weeks post-death; included HADS screening and a debrief with psychologist and physician. | High anxiety/depression symptoms among attendees; 86% rated program positively but low uptake (23%) | A phone call might be a first step to personalize bereavement support and identify needs for further meetings | Neutral |
|  | [34] | Follow-up offered by phone or in person; more frequent during early COVID wave; aimed to reflect on medical course and assess well-being | Many found conversations helpful to process loss and feel acknowledged; some unaware this option existed | ICU professionals may play a vital role by proactively offering follow-up; recommend inclusion in ICU guidelines | Positive |
|  | [35] | Families met with ICU physician and optionally nurse/social worker; focus on understanding cause of death and ICU experience | 91% supported continuation of the meetings; 80% understood the cause of death better; high satisfaction reported | Follow-up meetings are appreciated by families and improve understanding; physician presence deemed especially important | Positive |
|  | [4] | At end of support group meeting/memorial, relatives invited to attend further support meetings, or meetings with individual doctors/nurses are arranged if necessary. | Based on feedback, if it is identified at a relatives support meeting that a relative has specific medical questions, a subsequent meeting is arranged between the relative and consultant with a member of the bereavement group if the relatives so wishes. | The feedback we have received from relatives, nursing and medical staff suggests that the service we are providing is meeting many of the bereaved relatives needs. However, more structured evaluation and research is planned for the future to evaluate the service and what relatives want from it. | Positive |
| Follow-up phone call | [5] | Phone call within a few weeks/month of pt passing away, +/- phone call @ 3-6 months | Helpful by all who responded/completed evaluation. Additional comments from families noted to be positive. | Our Bereavement Follow-up program has been viewed as a success by both staff and families. We have shown how a group of staff nurses, given support and some minimal time away from direct patient care responsibilities, can develop and implement a program of follow-up after patient deaths. | Positive |
|  | [36] | A phone call two to three months after the loss by specialized nurse (standard of care at hospital) | The follow-up telephone call was rated as a little emotional, however this varies among the family members (6 [2–6]) and this call aroused both pleasant and unpleasant memories (4 [3–6]). | In general, the quality of care, and ‘end-of-life care’ in the ICU is good, as assessed by relatives of deceased ICU patients. To optimize the care and ‘end-of-life care’ in the ICU, improvements in terms of information provision and possibilities to visit the patient can be made. The follow-up calls were appreciated and beneficial but could be improved by identifying the relatives’ preferences for a scheduled or unscheduled conversation. | Neutral |
|  | [37] | 1 hour interview 1-3 years after patient death asking what they would have liked during/after time in the unit during bereavement. | Respondents asked how they coped after loss – one spoke of network of friends who helped, while half (8) talked of supportive family members who ‘were an enormous help’, and ‘very supportive’. Respondents asked about suggestions to make experience less traumatic - 10 (62.5%) mentioned a ‘counsellor’, ‘someone to talk to’, or ‘someone to express thoughts and feelings to’. 25% (4) of respondents would have liked someone to give advice on ‘organizing things’ (the funeral) or a ‘warning of the painful emotions’ which would follow | If nurses address the issues which have been highlighted and provide support networks not only for the relatives but also for colleagues, then the stress and uneasiness surrounding death may be dealt with in a more positive way in future. | Neutral |
|  | [9] | Follow-up phone call at 4 to 5 weeks - assess how doing and identify additional needs. A second follow-up phone call at 6 months to provide more opportunities for feedback. | No sig. difference in depression, anxiety, PTSD, and ICU satisfaction scores. Significantly more family members in non-bereavement group experienced prolonged grief (3 vs 0 participants). Many did not remember receiving follow-up telephone calls. 42% preferred that f/u be done by grief specialists. 47%. preferred that f/u only occur for 1 month after death. | Bereavement follow-up after an ICU death helped lower the risk of prolonged grief and may help lower the risk of PTSD in family members. It did not affect anxiety, depression, or satisfaction with care. The results regarding the most useful components of the bereavement program were mixed, but it was clear that ICU families do want this type of support. | Positive |
|  | [10] | Provided a folder (@ 4 weeks) with a letter noting that study author/bereavement committee would keep in touch for a year; phone call to the family at 6 weeks. | 158/160 responses expressed positive reactions to bereavement program. | Throughout the program, bereaved family members have responded positively, and the nursing staff in our busy unit continues to keep in touch with them. Our bereavement program is providing comfort for the families and closure for the nurses. | Positive |
|  | [3] | Telephone follow-up by social workers occurred 3 weeks after pt's death. | Among those who responded to the survey and responded they received a telephone call (64%; n=7/11), the majority (67%; n=4/6) found it helpful. When asked what they found helpful, they described that the verbal support gave them hope, and the call was a thoughtful and caring gesture. Overall, on a scale of 0–10, the mean rating for program helpfulness was 7 (range 5–10), with 44% rating the program 7 or higher | Our study shows that a formal, multi-component bereavement program for family members of patients who die in the ICU is feasible to implement, yet the value of each individual component of the program is family member dependent. The development of bereavement follow-up programs should take into consideration the resources available in the institution and the unique needs of individual families for optimal impact. | Positive |
|  | [14] | Semi-structured nurse-led call; allowed questions, offered follow-up, no fixed duration | Non-significant decrease in anxiety/depression scores (HADS); no effect on other outcomes | Bereavement calls did not significantly impact psychological outcomes at 6 months | Neutral |
|  | [13] | After death family member given a bereavement pack, which included notice of follow-up call planned for 6–8 weeks after the patient’s death. The bedside nurse highlighted to the family member the follow-up call and the letter, which included the option to decline participation in the project. | Family members’ feedback regarding the follow-up calls were almost universally positive. It was consistently reported that a telephone conversation was preferred over face-to-face interview. | The focus on quality of end-of-life care promoted recruitment and potentially identified previously missed possible donors. Bereavement telephone follow-up harnessed the insights of consumers to identify opportunities to improve endof-life care. The feasibility of this collaborative strategy should be further tested in other healthcare environments. | Positive |
| Training for staff | [29] | ICU FM toolkit (includes QI project syllabus, samples of ICU-FM note templates) 2) didactics and training (interactive webinars, interdisciplinary role-play to demonstrate key communication strategies and skills); and 3) networking and coaching. The toolkit included handouts, pocket cards, and work sheets for clinicians to reference | Bereaved Family Survey-Performance Measure (BFS-PM) score improved | A ICU-FM QI Initiative demonstrated an increase in documented FMs in VA medical center decedents, which is an important process measure of ICU care quality. | Positive |
|  | [12] | Management of the NCC required that all full-time and part-time patient care technicians and nursing staff participate in the training. A total of 5 repeat sessions (4 hours of content) were taught by members of the Embrace Hope team at various times of day to accommodate day and off-shift schedules and new hires. Nurse and technician training included content on the components of the Embrace Hope structured process, symptom onset/ management during terminal stages, communication techniques during grief, cultural considerations of dying, and institutional resources available to support end-of-life care. Physician education was 1 hour and included a brief overview of the Embrace Hope intervention and evidence-based symptom management given by the palliative care physician. | Decreased variability was noted by surrogates in regard to symptom control, communication, and emotional support provided by NCC staff. Statistically significant changes in staff members’ perceived ability to provide a “good death" and also statistically significant decrease in perceived barriers to their ability to provide end-of-life care. | Through hard work and emphasis on supporting patients and their families at the end of life, quality care can be provided within the walls of inpatient critical care units. Emphasizing the skill sets of an entire multidisciplinary team enables a structured intervention that optimizes caring while enabling a manageable workflow for team members. | Positive |
| Other | [38] | Six discrete ∼15-min modules. Utilizes empathic support, psychoeducation, and experiential exercises to reduce experiential avoidance, grief, anxiety, and peritraumatic distress. Two booster calls conducted (two and four weeks later) to review the key principles of EMPOWER, discuss successes and challenges, engage in coping rehearsal. Call also often provided acute meaning-centered bereavement support and reviews how to apply the EMPOWER principles in coping with post-loss grief and bereavement. | 100% of participants who began the intervention completed all 6 modules. High ratings of effectiveness and helpfulness. Results showed immediate post-intervention improvements in anxiety, peritraumatic distress, and experiential avoidance. At 3-month f/u improvements in prolonged grief symptoms, depression, anxiety, and experiential avoidance. | Preliminary data suggest that EMPOWER is feasible, acceptable, and associated with notable improvements in psychological symptoms among surrogates. Future research should examine EMPOWER with a larger sample in a randomized controlled trial. | Positive |
|  | [39] | A moment of silence (The Pause) observed at bedside immediately after death, with standardized respectful wording | Reported to reduce staff burnout and support families in early grief; families expressed appreciation via various channels | The Pause helps honor patients, supports grieving families, and reaffirms the values of care and compassion | Positive |
|  | [27] | Grieving cart: the secretary calls dietary staff, who prepare food trays to be picked up by ICU staff. Fresh coffee and tea brewed on the unit are poured into carafes when the bakery items arrive on the unit. Muffins, cookies, tea, coffee, and water are assembled on the cart. The top drawer of the cart contains English and Spanish versions of the Bible, Koran, and Book of Mormon and pamphlets about grief and bereavement. The lower portion of the cart holds paper cups, napkins, and condiments. | 100% (65/65) positive responses, included: ‘‘such a big place still cares about us", the cart "was a gesture that helps you understand that people care", ‘‘we were starving but didn’t want to leave the room to go to the cafeteria". | The grieving cart has been an inexpensive, low-tech intervention that has resulted in a profound change in our ICU philosophy and the nursing care delivered to families just beginning the grieving process. The grieving cart serves as a symbol of caring and support for families and a way for nurses to complete the circle of caring in which aggressive, cure driven care becomes palliative care and peaceful death. | Positive |

# eSupplement: Database Search Strategies

**CINAHL**: Inception - July 3, 2025


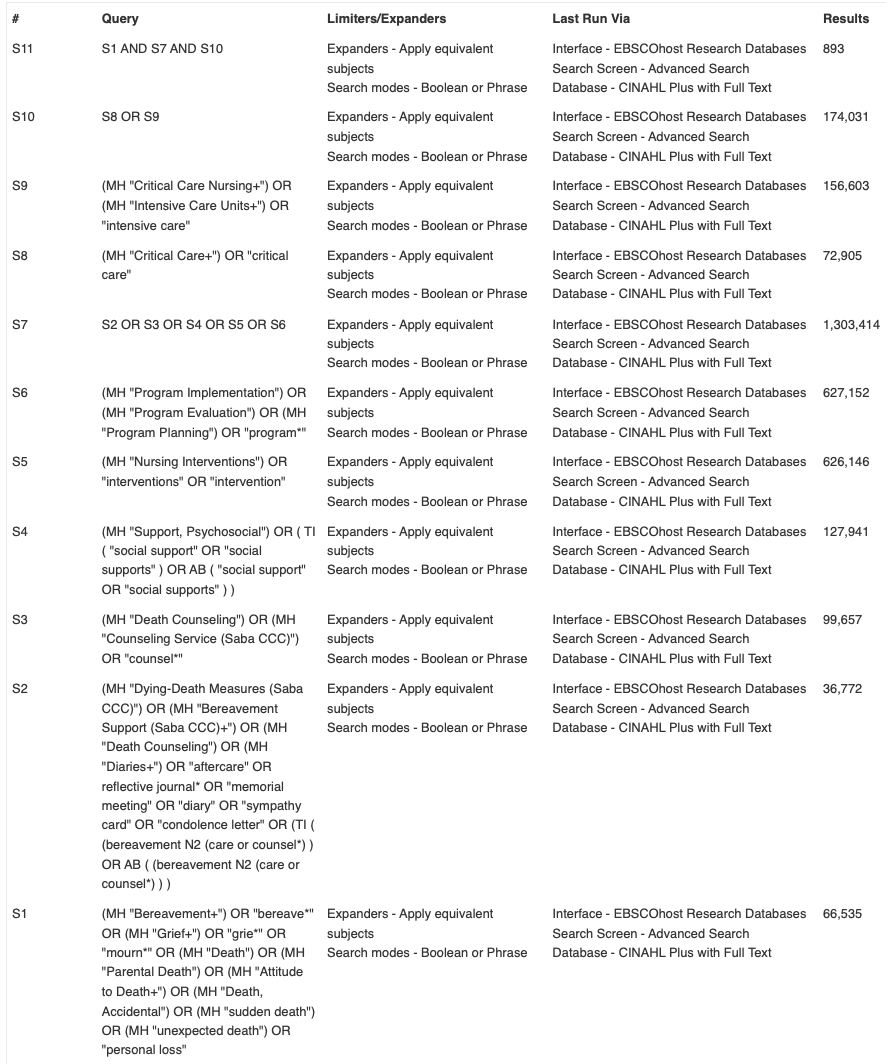


**APA PsycInfo**: Inception - July 3, 2025


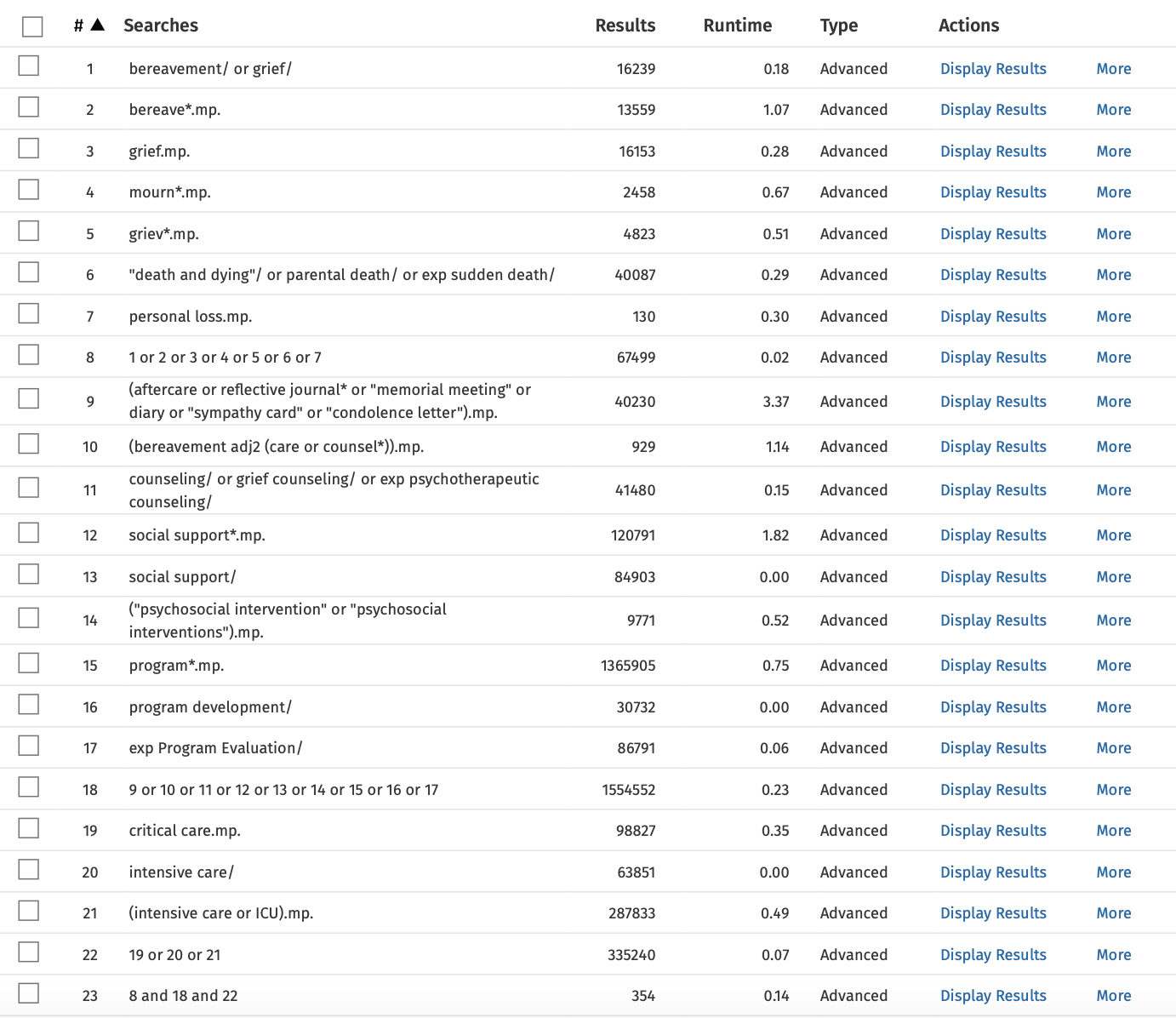


**Web of Science**: Inception - July 3, 2025

1. TS=(bereavement OR bereave)
2. TS=(grief OR grieving)
3. TS=(mourn*)
4. TS=(death OR "sudden death" OR "unexpected death")
5. TS=("personal loss")
6. #1 OR #2 OR #3 OR #4 OR #5
7. TS=(aftercare OR "reflective journal" OR "memorial meeting" OR "diary" OR "diaries" OR "sympathy card" OR "condolence letter")
8. TS=(bereavement NEAR/2 (care OR counsel*))
9. TS=("social support" OR "social supports")
10. TS=("psychosocial intervention" OR "psychosocial interventions")
11. TS=(program* OR "program development" OR "program evaluation")
12. #8 OR #9 OR #10 OR #11
13. TS=(critical care OR intensive care OR ICU)
14. #6 AND #7 AND #12 AND #13

**MEDLINE:** Inception – July 3, 2025


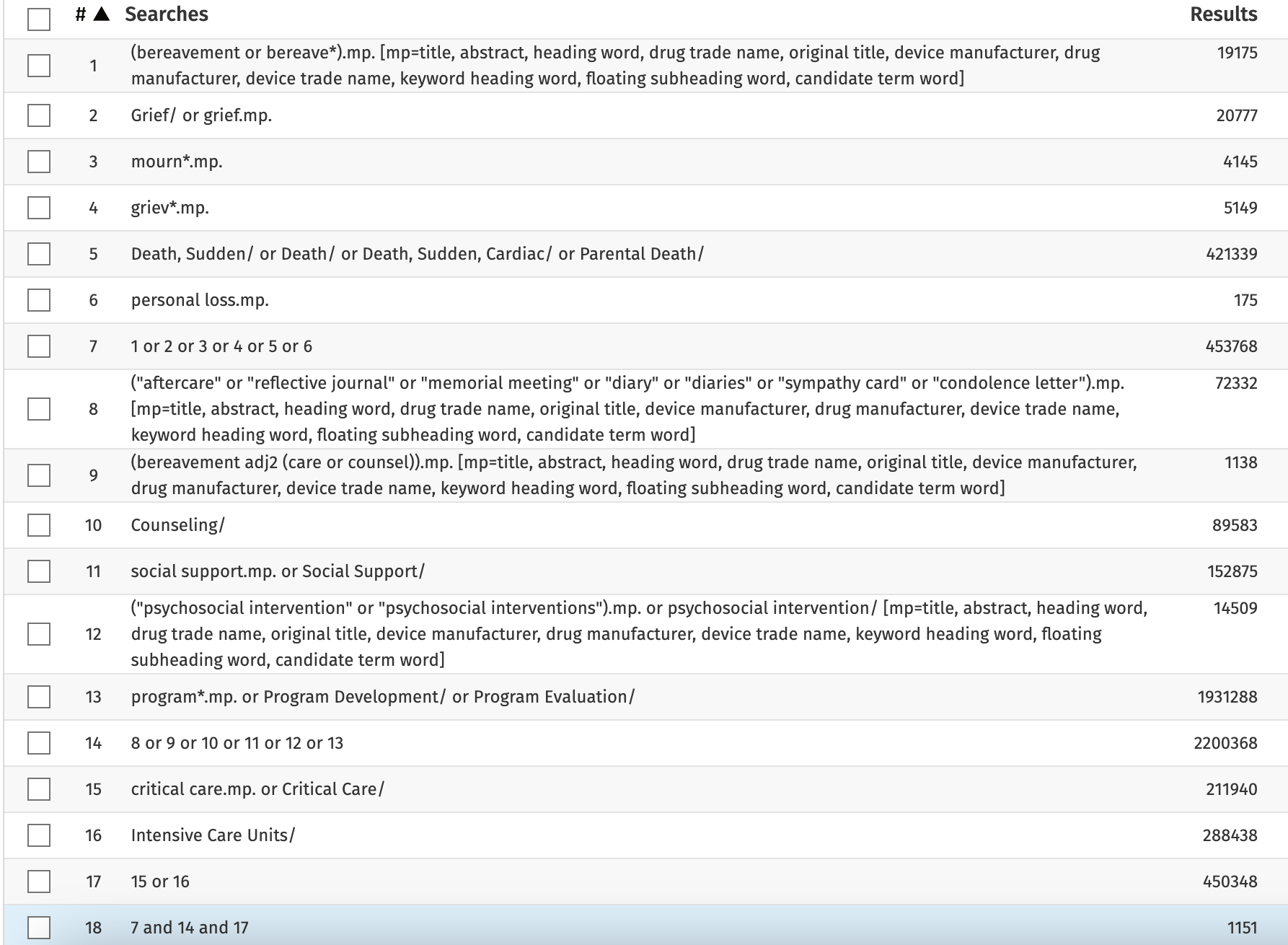


**EMBASE**: Inception - July 3, 2025


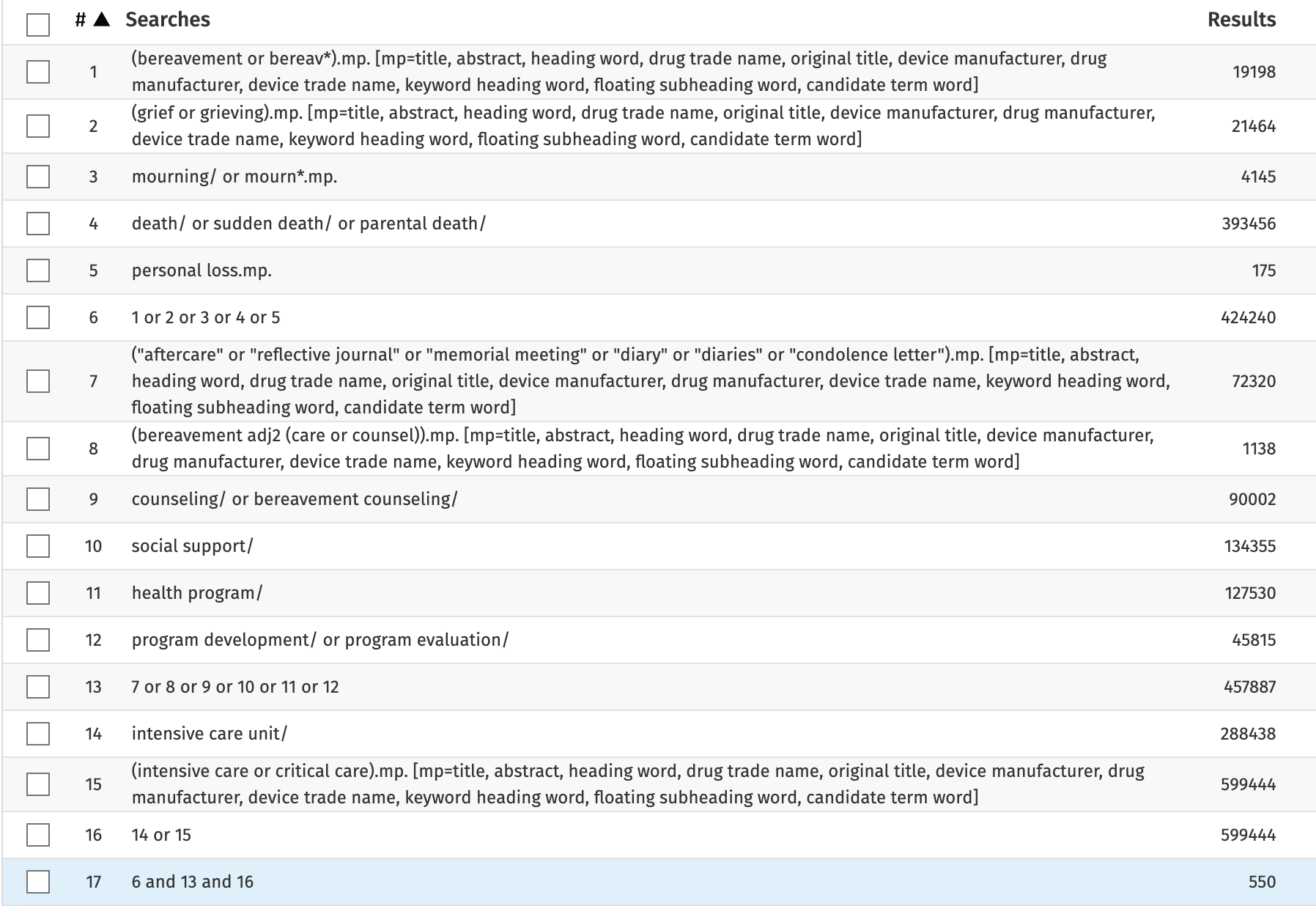


# References

1. Harris D, Polgarova P, Enoch L. Service evaluation of the bereavement care delivered in a UK intensive care unit. British Journal of Nursing [Internet]. 2021;30(11):644–50. Available from: http://dx.doi.org/10.12968/bjon.2021.30.11.644

2. Platt J. The planning, organising and delivery of a memorial service in critical care. Nurs Crit Care [Internet]. 2004;9(5):222–9. Available from: http://cyber.usask.ca/login?url=https://search.ebscohost.com/login.aspx?direct=true&db=rzh&AN=106560203&site=ehost-live

3. Santiago C, Lee C, Piacentino R, Deveau C, Villeneuve J, Diston MT, et al. A pilot study of an interprofessional, multicomponent bereavement follow-up program in the intensive care unit. Canadian Journal of Critical Care Nursing [Internet]. 2017;28(3):18–24. Available from: http://cyber.usask.ca/login?url=https://search.ebscohost.com/login.aspx?direct=true&db=rzh&AN=125473168&site=ehost-live

4. Williams R, Harris S, Randall L, Nichols R, Brown S. A bereavement after‐care service for intensive care relatives and staff: the story so far. Nurs Crit Care [Internet]. 2003;8(3):109–15. Available from: http://dx.doi.org/10.1046/j.1478-5153.2003.00017.x

5. Anderson AH, Bateman LH, Ingallinera KL, Woolf PJ. Our caring continues: a bereavement follow-up program. Focus Crit Care [Internet]. 1991;18(6):523–6. Available from: http://cyber.usask.ca/login?url=https://search.ebscohost.com/login.aspx?direct=true&db=rzh&AN=107481549&site=ehost-live

6. Erikson A, Puntillo K, McAdam J. Family members’ opinions about bereavement care after cardiac intensive care unit patients’ deaths. Nurs Crit Care [Internet]. 2019;24(4):209–21. Available from: http://dx.doi.org/10.1111/nicc.12439

7. Kentish-Barnes N, Cohen-Solal Z, Souppart V, Galon M, Champigneulle B, Thirion M, et al. “It Was the Only Thing I Could Hold Onto, But…”: Receiving a Letter of Condolence After Loss of a Loved One in the ICU: A Qualitative Study of Bereaved Relatives’ Experience*. Crit Care Med [Internet]. 2017;45(12):1965–71. Available from: http://dx.doi.org/10.1097/ccm.0000000000002687

8. Kentish-Barnes N, Chevret S, Champigneulle B, Thirion M, Souppart V, Gilbert M, et al. Effect of a condolence letter on grief symptoms among relatives of patients who died in the ICU: a randomized clinical trial. Intensive Care Med. 2017 Apr 1;43(4):473–84.

9. McAdam JL, Puntillo K. Pilot study assessing the impact of bereavement support on families of deceased intensive care unit patients. American Journal of Critical Care. 2018 Sep 1;27(5):372–80.

10. Ross MW. In our unit. Implementing a bereavement program. Crit Care Nurse [Internet]. 2008;28(6):87–8. Available from: http://cyber.usask.ca/login?url=https://search.ebscohost.com/login.aspx?direct=true&db=rzh&AN=105587903&site=ehost-live

11. Takaoka A, Vanstone M, Neville TH, Goksoyr S, Swinton M, Clarke FJ, et al. Family and Clinician Experiences of Sympathy Cards in the 3 Wishes Project. American Journal of Critical Care [Internet]. 2020;29(6):422–8. Available from: http://cyber.usask.ca/login?url=https://search.ebscohost.com/login.aspx?direct=true&db=rzh&AN=146653053&site=ehost-live

12. Yeager S, Doust C, Epting S, Iannantuono B, Indian C, Lenhart B, et al. Embrace Hope: An End-of-Life Intervention to Support Neurological Critical Care Patients and Their Families. Crit Care Nurse [Internet]. 2010;30(1):47–58. Available from: http://dx.doi.org/10.4037/ccn2010235

13. Yeo NYK, Reddi B, Kocher M, Wilson S, Jastrzebski N, Duncan K, et al. Collaboration between the intensive care unit and organ donation agency to achieve routine consideration of organ donation and comprehensive bereavement follow-up: an improvement project in a quaternary Australian hospital. Australian Health Review [Internet]. 2021;45(1):124. Available from: http://dx.doi.org/10.1071/ah20005

14. Showler L, Rait L, Chan M, Tondello M, George A, Tascone B, et al. Communication with bereaved family members after death in the ICU: the CATHARTIC randomised clinical trial. Critical Care and Resuscitation. 2022 Jun 1;24(2):116–27.

15. Azad MA, Swinton M, Clarke FJ, Takaoka A, Vanstone M, Woods A, et al. Experiences of Bereaved Family Members Receiving Commemorative Paintings: A Qualitative Study. JAMA Netw Open [Internet]. 2020 Dec 1;3(12):e2027259–e2027259. Available from: https://pubmed.ncbi.nlm.nih.gov/33346843

16. Beiermann M, Kalowes P, Dyo M, Mondor A. Family Members’ and Intensive Care Unit Nurses’ Response to the ECG Memento© during the Bereavement Period. Dimensions of Critical Care Nursing. 2017 Nov 1;36(6):317–26.

17. Neville TH, Clarke F, Takaoka A, Sadik M, Vanstone M, Phung P, et al. Keepsakes at the End of Life. J Pain Symptom Manage [Internet]. 2020;60(5):941–7. Available from: http://cyber.usask.ca/login?url=https://search.ebscohost.com/login.aspx?direct=true&db=rzh&AN=146535422&site=ehost-live

18. Riegel M, Buckley T, Randall S. Family’s experience of memory making in adult intensive care and its use in early bereavement: A descriptive qualitative study. J Clin Nurs. 2023 Sep 1;32(17–18):6648–61.

19. Combe D. The use of patient diaries in an intensive care unit. Nurs Crit Care [Internet]. 2005;10(1):31–4. Available from: http://dx.doi.org/10.1111/j.1362-1017.2005.00093.x

20. Johansson M, Wåhlin I, Magnusson L, Runeson I, Hanson E. Family members’ experiences with intensive care unit diaries when the patient does not survive. Scand J Caring Sci [Internet]. 2017;32(1):233–40. Available from: http://dx.doi.org/10.1111/scs.12454

21. Melby AC, Litleré Moi A, Gjengedal E. The experiences of bereaved relatives on receiving the intensive care diaries of their loved ones. Norwegian Journal of Clinical Nursing / Sykepleien Forskning [Internet]. 2020;1–17. Available from: http://cyber.usask.ca/login?url=https://search.ebscohost.com/login.aspx?direct=true&db=rzh&AN=146641421&site=ehost-live

22. Schoeman T, Sundararajan K, Micik S, Sarada P, Edwards S, Poole A, et al. The impact on new-onset stress and PTSD in relatives of critically ill patients explored by diaries study (The “INSPIRED” study). AUSTRALIAN CRITICAL CARE. 2018;31(6):382–9.

23. Bazzano G, Buccoliero F, Villa M, Pegoraro F, Iannuzzi L, Rona R, et al. The role of intensive care unit diaries in the grieving process: A monocentric qualitative study. Nurs Crit Care. 2024 Jul 1;29(4):706–14.

24. Barnato AE, Schenker Y, Tiver G, Dew MA, Arnold RM, Nunez ER, et al. Storytelling in the Early Bereavement Period to Reduce Emotional Distress Among Surrogates Involved in a Decision to Limit Life Support in the ICU: A Pilot Feasibility Trial. Crit Care Med [Internet]. 2017;45(1):35–46. Available from: http://cyber.usask.ca/login?url=https://search.ebscohost.com/login.aspx?direct=true&db=rzh&AN=120332987&site=ehost-live

25. Schenker Y, Dew MA, Reynolds CF, Arnold RM, Tiver GA, Barnato AE. Development of a post-intensive care unit storytelling intervention for surrogates involved in decisions to limit life-sustaining treatment. Palliat Support Care. 2013 Jun 13;13(3):451–63.

26. Takaoka A, Honarmand K, Vanstone M, Tam B, Smith OM, Baker A, et al. Organ Donation at the End of Life: Experiences From the 3 Wishes Project. J Intensive Care Med [Internet]. 2020;36(4):404–12. Available from: http://dx.doi.org/10.1177/0885066619900125

27. Whitmer M, Hurst S, Stadler K, Ide R. Caring in the curing environment: the implementation of a grieving cart in the ICU. Journal of Hospice & Palliative Nursing [Internet]. 2007;9(6):329–33. Available from: http://cyber.usask.ca/login?url=https://search.ebscohost.com/login.aspx?direct=true&db=rzh&AN=105970797&site=ehost-live

28. Neville TH, Taich Z, Walling AM, Bear D, Cook DJ, Tseng CH, et al. The 3 Wishes Program Improves Families’ Experience of Emotional and Spiritual Support at the End of Life. J Gen Intern Med. 2023 Jan 1;38(1):115–21.

29. Akgün KM, Gruenewald DA, Smith D, Wertheimer D, Luhrs C. A National VA Palliative Care Quality Improvement Project for Improving Intensive Care Unit Family Meetings (ICU-FMs). J Pain Symptom Manage [Internet]. 2019;58(6):1075–80. Available from: http://cyber.usask.ca/login?url=https://search.ebscohost.com/login.aspx?direct=true&db=rzh&AN=141607907&site=ehost-live

30. Kentish-Barnes N, Chevret S, Valade S, Jaber S, Kerhuel L, Guisset O, et al. A three-step support strategy for relatives of patients dying in the intensive care unit: a cluster randomised trial. The Lancet. 2022 Feb 12;399(10325):656–64.

31. Mosenthal AC, Murphy PA, Barker LK, Lavery R, Retano A, Livingston DH. Changing the culture around end-of-life care in the trauma intensive care unit. J Trauma. 2008;64(6):1587–93.

32. Poppe C, Akum S, Crombez G, Rogiers X, Hoste E. Evaluation of the quality of the communication and emotional support during the donation procedure: The use of the donor family questionnaire (DFQ). J Crit Care. 2019;53:198–206.

33. Dekeyser T, Sejourné C, Marzouk M, Rahmani I, Vinsonneau C. Combined psychologist-physician post-death meeting as part of an integrated bereavement program for families. Vol. 47, Intensive Care Medicine. Springer Science and Business Media Deutschland GmbH; 2021. p. 795–7.

34. Renckens SC, Onwuteaka-Philipsen BD, Jorna Z, Klop HT, du Perron C, van Zuylen L, et al. Experiences with and needs for aftercare following the death of a loved one in the ICU: a mixed-methods study among bereaved relatives. BMC Palliat Care. 2024 Dec 1;23(1).

35. Kock M, Berntsson C, Bengtsson A. A follow-up meeting post death is appreciated by family members of deceased patients. Acta Anaesthesiol Scand. 2014;58(7):891–6.

36. Brekelmans ACM, Ramnarain D, de Haas M, Ruitinga R, Pouwels S. Evaluation of ICU end-of-life and bereavement care by relatives of deceased ICU patients. Respir Med [Internet]. 2022;202:106972. Available from: http://dx.doi.org/10.1016/j.rmed.2022.106972

37. Hall B, Hall DA. Learning from the experience of loss: people bereaved during intensive care. Intensive Crit Care Nurs [Internet]. 1994;10(4):265–70. Available from: http://dx.doi.org/10.1016/0964-3397(94)90035-3

38. Lichtenthal WG, Viola M, Rogers M, Roberts KE, Lief L, Cox CE, et al. Development and preliminary evaluation of EMPOWER for surrogate decision-makers of critically ill patients. Palliat Support Care [Internet]. 2022;20(2):167–77. Available from: http://cyber.usask.ca/login?url=https://search.ebscohost.com/login.aspx?direct=true&db=rzh&AN=156712296&site=ehost-live

39. Pinnington M, Westwood A. Introducing the pause after the death of a patient in critical care. Nursing times (1987). 2024;120(3):30–1.
